# Flexible and Waterproof 2D/1D/0D Construction of MXene-Based Nanocomposites for Electromagnetic Wave Absorption, EMI Shielding, and Photothermal Conversion

**DOI:** 10.1007/s40820-021-00673-9

**Published:** 2021-06-25

**Authors:** Zhen Xiang, Yuyang Shi, Xiaojie Zhu, Lei Cai, Wei Lu

**Affiliations:** Shanghai Key Lab of D&A for Metal-Functional Materials, School of Materials Science & Engineering, Tongji University, Shanghai, 201804 People’s Republic of China

**Keywords:** Ti_3_C_2_T_x_, CNTs, Co, Low-dimensional materials, Electromagnetic wave absorption, EMI shielding, Multifunction

## Abstract

**Abstract:**

High-performance electromagnetic wave absorption and electromagnetic interference (EMI) shielding materials with multifunctional characters have attracted extensive scientific and technological interest, but they remain a huge challenge. Here, we reported an electrostatic assembly approach for fabricating 2D/1D/0D construction of Ti_3_C_2_T_x_/carbon nanotubes/Co nanoparticles (Ti_3_C_2_T_x_/CNTs/Co) nanocomposites with an excellent electromagnetic wave absorption, EMI shielding efficiency, flexibility, hydrophobicity, and photothermal conversion performance. As expected, a strong reflection loss of -85.8 dB and an ultrathin thickness of 1.4 mm were achieved. Meanwhile, the high EMI shielding efficiency reached 110.1 dB. The excellent electromagnetic wave absorption and shielding performances were originated from the charge carriers, electric/magnetic dipole polarization, interfacial polarization, natural resonance, and multiple internal reflections. Moreover, a thin layer of polydimethylsiloxane rendered the hydrophilic hierarchical Ti_3_C_2_T_x_/CNTs/Co hydrophobic, which can prevent the degradation/oxidation of the MXene in high humidity condition. Interestingly, the Ti_3_C_2_T_x_/CNTs/Co film exhibited a remarkable photothermal conversion performance with high thermal cycle stability and tenability. Thus, the multifunctional Ti_3_C_2_T_x_/CNTs/Co nanocomposites possessing a unique blend of outstanding electromagnetic wave absorption and EMI shielding, light-driven heating performance, and flexible water-resistant features were highly promising for the next-generation intelligent electromagnetic attenuation system.
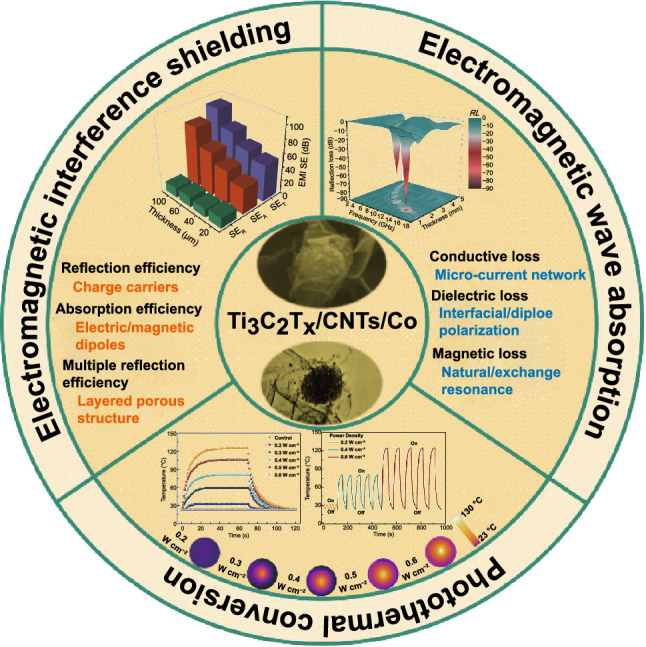

**Highlights:**

- The 2D/1D/0D Ti_3_C_2_T_x_/carbon nanotubes/Co nanocomposite is successfully synthesized via an electrostatic assembly.
- Nanocomposites exhibit an excellent electromagnetic wave absorption and a remarkable electromagnetic interference shielding efficiency.
- The flexible, waterproof, and photothermal conversion performances are achieved.

**Supplementary Information:**

The online version contains supplementary material available at 10.1007/s40820-021-00673-9.

## Introduction

The rapid advancement of communication and increasingly compact and intelligent electronic devices have caused serious electromagnetic interference (EMI), information leakage, and even affected human health and the surrounding environment [1]. Generally, an effective electromagnetic attenuation material can reduce the reflection and transmission of undesirable electromagnetic waves. In addition, it is highly desirable to integrate lightweight, thin thickness, and flexibility into one material for the next-generation electromagnetic wave absorbing and EMI shielding applications [2–4].

Low-dimensional nanomaterials owing to their distinctive properties of large surface area, flexibility, and tunable electronic structure have been receiving great attention [5–8]. MXene (multifunctional two-dimensional (2D) layered metal carbides and nitrides) has a great potential in novel electromagnetic wave absorption and EMI shielding materials due to its unique multilayer microstructure, high specific surface area, good electrical conductivity, and metal-like properties [9–12]. For the electromagnetic wave absorption material, it was characterized by the little reflection and transmission of the incident electromagnetic wave. The moderated impedance matching and electromagnetic parameters were required to achieve the more capture and efficient attenuation of electromagnetic waves. In addition, the high electric conductivity and multiple internal reflections in MXene contributed to the formation of high-efficient EMI shielding materials [10]. Thus, it is necessary to explore the development of the electromagnetic wave absorption and EMI shielding materials by making full use of the synergy of the component loss mechanism and rationally designing a novel architecture. Recently, several materials, including intrinsic conductive polymers, magnetic nanoparticles/nanowires, and carbon nanomaterials of graphene sheets and carbon nanotubes, have been strategically combined with MXene to explore the novel electromagnetic attenuation materials. Wang et al*.* prepared flexible and lightweight Ti_3_C_2_T_x_/Fe_3_O_4_@PANI composite, which achieved a high-performance EMI shielding performance [13]. Wang et al*.* reported that hierarchical Ti_3_C_2_T_x_ MXene/Ni Chain/ZnO on cotton fabric showed self-cleaning and improved microwave absorption [14]. Wang et al*.* prepared hierarchical carbon fiber@MXene@MoS_2_ composites with an efficient electromagnetic wave absorption [15]. Li et al*.* manufactured Ti_3_C_2_T_x_ MXene@graphene oxide aerogel microspheres by rapid freezing assisted electrostatic-spinning, which exhibited a low filler loading, a thin thickness, and a strong reflection loss [16]. Among them, one-dimensional (1D) carbon nanotubes (CNTs) with high conductivity, mechanical reliability, lightweight were integrated with MXene to assemble MXene/CNTs composites for efficiently shielding the electromagnetic waves [4, 17, 18] and absorbing electromagnetic wave [19]. Moreover, due to their high Snoek’s limit, conductivity, strong anisotropy field, and high saturation magnetization, zero-dimensional (0D) magnetic nanoparticles (MNP) were widely used in electromagnetic wave absorbing and EMI shielding [20–24]. Thus, combining magnetic nanoparticles with MXene can achieve enhanced electromagnetic dissipation materials, such as MXene/hollow Fe_3_O_4_ [25], TiO_2_/Ti_3_C_2_T_x_/Fe_3_O_4_ [26], MXene/Ni chain hybrid [27], Ti_3_C_2_T_x_@NiCo_2_O_4_ [28]. The 0D magnetic nanoparticles decorated 1D CNTs can be derived from the metal–organic frameworks (MOFs) due to their tunable composition and microstructure [29–31]. In addition, substantial development of electromagnetic wave absorption and EMI shielding was achieved for MXene-based composites with a novel structure. Examples included hollow structures [32–35], porous structures [36, 37, 2], and layer structures [38, 39]. The laminated porous structure gives the material the advantages of a lightweight, abundant interfacial polarization, and multiple scattering and reflection, which can attenuate more electromagnetic wave energy [40–42]. However, there were few reports on the laminated porous structure with the combination of 0D magnetic nanoparticles, 1D CNTs, and 2D MXene for the high-efficiency electromagnetic wave absorption and EMI shielding. Therefore, on the basis of the complementarity in composition and structure, combining 0D magnetic nanoparticles, 1D CNTs, and 2D MXene into the laminated MXene/CNTs/MNP nanocomposites is worthy of extensive research toward high-efficiency electromagnetic attenuation materials.

Furthermore, with the popularity of emerging highly integrated fifth-generation (5G) wireless technologies and wearable devices, the electromagnetic wave absorption and EMI shielding materials have been strikingly updated by integrating their inherent absorbing and shielding capabilities as well as novel functionalities, including flexible, hydrophobic, and energy conversion functions [43]. Examples such as flexible Fe_3_O_4_@Ti_3_C_2_T_x_/elastomer [44], MXene foam [37], PET-PPy/MXene textiles [45], CNF@MXene films [46], and MXene/Ag nanowire-PVA films [47]. Considering abundant surface termination (O, F, and/or OH groups) introduced during etching and delamination processes and the use under a humid condition, the degradation/oxidation of the MXene was easily proceeded in a high humidity environment, which may deteriorate their stability and reliability of electromagnetic wave absorption and EMI shielding performances. Thus, the water-resistant treatment of the surface is particularly important and urgently needed. Furthermore, light-to-heat, also known as a photothermal conversion, is an energy conversion process that harvests light energy by photothermal materials and converts it into thermal energy [48]. The efficient photothermal performance would broaden the practical applications range of MXene-based composites. It is of great significance to explore the photo-responsive behavior of MXene/CNTs/MNP nanocomposites. Therefore, the development of fabrication and functionalization for hydrophobic surfaces and photothermal conversion would be profitable in improving their practicability for electromagnetic wave absorption and EMI shielding in various technological applications.

Here, we demonstrated an electrostatic assembly approach for fabricating 2D/1D/0D construction of Ti_3_C_2_T_x_/CNTs/Co nanocomposites with highly integrated functions, including excellent electromagnetic wave absorption, EMI shielding efficiency, photothermal conversion performance, flexible and hydrophobic characterizations. The sea urchin-like CNTs/Co nanocomposites were introduced on 2D Ti_3_C_2_T_x_ MXene sheets to form laminated Ti_3_C_2_T_x_/CNTs/Co nanocomposites to improve the electromagnetic wave absorption and enhance the EMI shielding efficiency. As expected, a strong reflection loss of −85.8 dB, an ultrathin thickness of 1.4 mm, an ultralow filler loading of 5 wt%, and a broad EAB of 6.1 GHz were obtained. At the same time, the EMI shielding efficiency was as high as 110.1 dB. The corresponding mechanisms were discussed in detail. Moreover, a thin layer of polydimethylsiloxane rendered the hydrophilic hierarchical Ti_3_C_2_T_x_/CNTs/Co hydrophobic with a water contact angle of ~ 110.3°, which can prevent the degradation/oxidation of the MXene-based composites in high humidity conditions. The photothermal conversion performances of the multifunctional film and its thermal cycle stability and adjustability were also investigated.

## Experimental Section

### Materials

Cobalt(II) nitrate hexahydrate (Co(NO_3_)_2_·6H_2_O), adenine [C_5_H_5_N_5_], ethanol (C_2_H_6_O), N, N-dimethylformamide [HCON(CH_3_)_2_, DMF], concentrated hydrochloric acid (HCl, 12 M), lithium fluoride (LiF), hexadecyl trimethyl ammonium bromide (CTAB), and polydimethylsiloxane (PDMS) were purchased from Shanghai Aladdin Co., Ltd and used without further purification. Ti_3_AlC_2_ powder was purchased from Foshan XinXi Technology Co., Ltd.

### Preparation of Ti_3_C_2_T_x_/CNTs/Co Nanocomposites

**Scheme 1:**
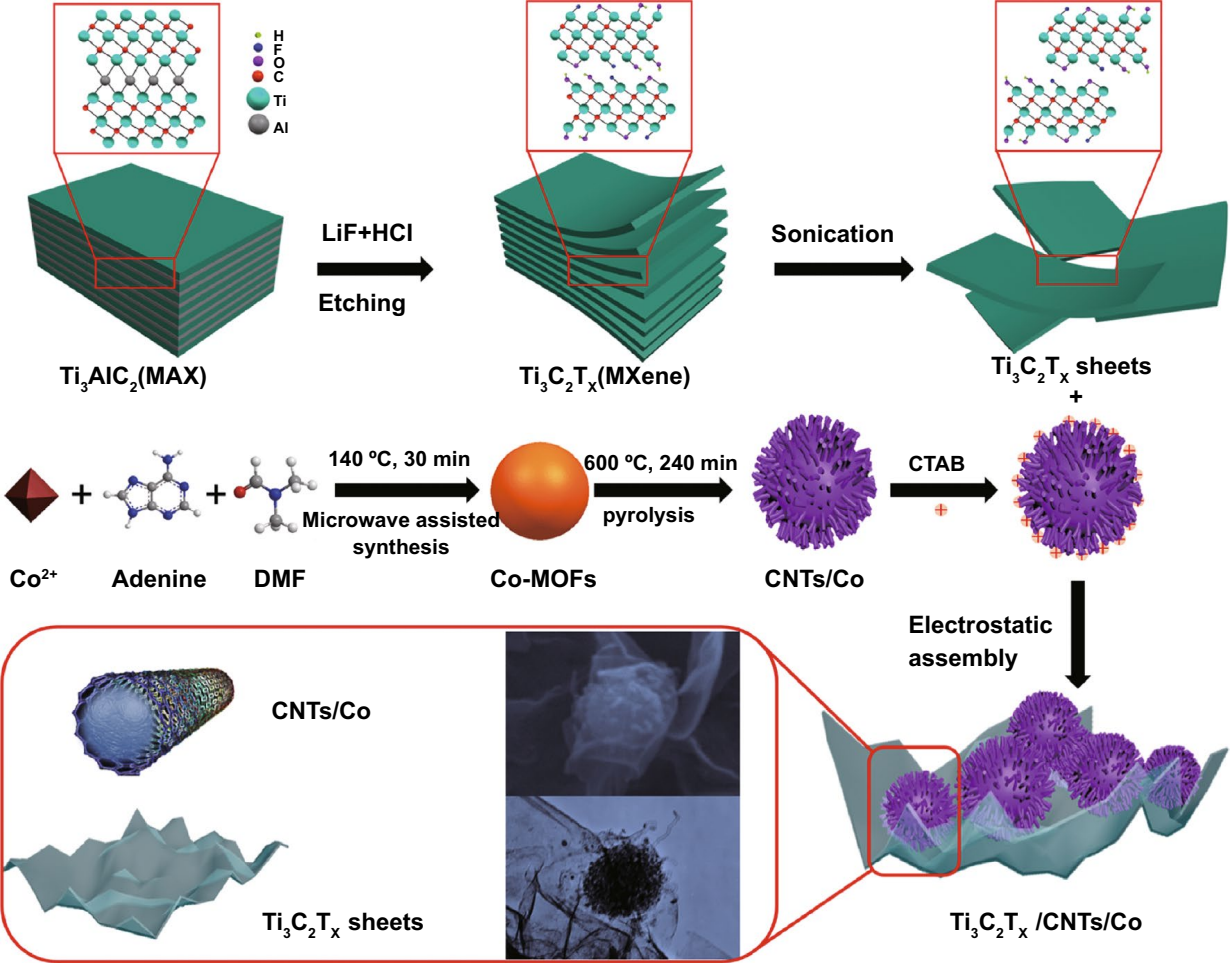
Schematic illustration of the Ti_3_C_2_T_x_/CNTs/Co nanocomposites formation process

The Ti_3_C_2_T_x_/CNTs/Co nanocomposites were synthesized as shown in Scheme 1. First, Ti_3_C_2_T_x_ sheets were prepared by the revised method [49, 50]. 1 g of LiF was added to 30 mL HCl solution (9 M) and stirred for 10 min with a magnetic Teflon stir bar to completely dissolve. 1 g of Ti_3_AlC_2_ powders were carefully added within 5 min and keep the reaction mixture at 40 °C for 24 h. The mixture was washed by deionized water and centrifuged (3,500 rpm, 5 min for each cycle) until the pH of the supernatant reached 5.5. The obtained sample was dispersed in 50 mL deionized water, followed by sonication at the temperature of 4 °C for 60 min in Ar atmosphere. The dark green Ti_3_C_2_T_x_ sheets solution was collected supernatant by centrifuging mixture at 3,500 rpm for 75 min. Secondly, 0.5 mM Co(NO_3_)_2_·6H_2_O and 1 mM adenine were dissolved in 50 mL DMF and magnetic stirring for 60 min. The resulting solution was transferred into a three-necked flask (100 mL) and then heated up to 140 °C for 30 min with a heating rate of 20 °C min^−1^, and then washed with DMF and ethanol, and dried at 60 °C overnight. The sea urchin-like CNTs/Co nanocomposites were prepared by pyrolysis of Co-MOFs template in a tube furnace at 600 °C for 240 min at a heating rate of 5 °C min^−1^ under H_2_:Ar (5:95 in volume %) flow. Thirdly, 10 mg CNTs/Co nanocomposites were dispersed in 50 mL CTAB solution (2 mg mL^−1^) by mechanical stirring for 30 min. Subsequently, 10 mL Ti_3_C_2_T_x_ sheets solution (1 mg mL^−1^) was added dropwise into the above mixture, and the mechanical stirring was continued for 60 min. Finally, the Ti_3_C_2_T_x_/CNTs/Co nanocomposites were collected by washing with deionized water by centrifugation, and then vacuum freeze-dried at −80 °C for 48 h. A PDMS solution was prepared by mixing elastomer and curing agent in a mass ratio of 10:1 at room temperature. The PDMS solution was evenly coated on the surface of Ti_3_C_2_T_x_/CNTs/Co nanocomposites with a coating rod and then cured in a vacuum oven at 80 °C for 4 h.

### Characterization

The morphology and microstructure of the synthesized samples were observed by scanning electron microscopy (SEM) and transmission electron microscopy (TEM). X-ray powder diffraction (XRD, Cu-Kα radiation) was used to analyze the crystallographic structure and phase composition of the samples. Raman spectroscopic system (633 nm laser excitation) was used to measure the Raman spectra. The room-temperature magnetic properties were tested by vibrating sample magnetometer (VSM). Nitrogen adsorption and desorption isotherms were tested by the Quad-resorb-SI instrument. The electrical conductivity of the samples was measured by using an advanced four-probe. The optical contact angle (CA) of the composite fabric was measured by the optical CA measurement system. An 808 nm high-power multimode pump laser was used as the NIR light source with a spot radius of about 6 mm. The thermal image and temperature of the samples were recorded by an infrared thermal imaging instrument.

### Electromagnetic Wave Absorption Measurements

Electromagnetic parameters were measured on a vector network analyzer (VNA, 3672B-S, Ceyear, China) in the frequency range of 2–18 GHz by a transmission–reflection mode. The obtained products with a loading of 5 wt% were uniformly mixed with paraffin, and pressed into coaxial rings (*Φ*_out_: 7.00 mm, *Φ*_in_: 3.04 mm). The reflection loss (*RL*) of Ti_3_C_2_T_x_, CNTs/Co, and Ti_3_C_2_T_x_/CNTs/Co nanocomposites was calculated by transmission line theory [51, 52]:

1 $${\text{RL}} = 20\log _{{10}} \left| {\frac{{Z_{{{\text{in}}}} - Z_{{\text{0}}} }}{{Z_{{{\text{in}}}} + Z_{0} }}} \right|$$

2 $$Z_{{{\text{in}}}} = Z_{0} (\mu _{r} /\varepsilon _{r} )^{{1/2}} \tanh \left[ {j\left( {2\pi fd/c} \right) \times \left( {\mu _{r} \cdot \varepsilon _{r} } \right)^{{1/2}} } \right]$$

where *Z*_0_, *Z*_in_, *f*, *c,* and *d* were the free space impedance, input impedance, frequency, light speed, and matching thickness of the absorber. The input impedance (|*Z*_in_/*Z*_0_|) was described as follows [53]:

3 $$\left| {Z_{{{\text{in}}}} /Z_{0} } \right| = |(\mu _{r} /\varepsilon _{r} )^{{1/2}} \tanh \left[ {j\left( {2\pi fd/c} \right) \times \left( {\mu _{r} \cdot \varepsilon _{r} } \right)^{{1/2}} } \right]|$$

The attenuation constant *α* was introduced [54, 55]

4 $$= \frac{{\sqrt 2 f}}{c} \times \sqrt {(^{{''}} \varepsilon ^{\prime\prime} - ^{'} \varepsilon ^{\prime}) + \sqrt {(^{{''}} \varepsilon ^{\prime\prime} - ^{'} \varepsilon ^{\prime})^{2} + (^{'} \varepsilon ^{\prime\prime} + ^{{''}} \varepsilon ^{\prime})^{2} } }$$

### EMI Shielding Measurements

EMI shielding effectiveness (*SE*) of all the samples was measured by a rectangular waveguide (32,117) using a 2-port network analyzer (3672B-S, Ceyear, China) in the frequency range of 8.2–12.4 GHz. A film with a diameter of 40 mm was obtained by a vacuum filtration method. Specifically, the measured volume dispersion of the MXene and CNTs/Co with a mass ratio (90:10, 80:20, 70:30, and 60:40) was filtered through a PES membrane with a pore size of 0.22 μm. The EMI *SE* of all the samples was calculated using *S*-parameters. The total EMI *SE* (*SE*_total_), consisting of reflection efficiency (*SE*_R_), absorption efficiency (*SE*_A_), and multiple reflection efficiency (*SE*_MR_), can be written as:

5 $${\text{SE}}_{{{\text{total}}}} = {\text{SE}}_{R} + {\text{SE}}_{A} + {\text{SE}}_{{{\text{MR}}}}$$

where SE_MR_ is often negligible when SE_total_ > 15 dB. SE_R_ and SE_A_ were expressed as reflection and absorption coefficient considering the power of the incident electromagnetic wave inside the shielding material as:

6 $${\text{SE}}_{R} = 10{\text{ }}\log \left(\frac{1}{{1 - \left| {S_{{11}} } \right|^{2} }}\right)$$

7 $${\text{SE}}_{A} = 10{\text{ }}\log {\text{ }}\left(\frac{{1 - \left| {S_{{11}} } \right|^{2} }}{{\left| {S_{{21}} } \right|^{2} }}\right)$$

According to Simon’s formula, the *SE*_T_ can be written as [9]:

8 $${\text{SE}}_{T} = 50 + 10\log \left( {\frac{\sigma }{f}} \right) + 1.7d\sqrt f$$

where *σ*, *f*, and *d* were the electrical conductivity, frequency, and thickness of the shielding materials, respectively.

### Photothermal Performance Measurements

The photothermal performance of the samples was studied under an 808 nm NIR laser with different power densities (0.2, 0.3, 0.4, 0.5, and 0.6 W cm^−2^). The photothermal conversion efficiency (*η*) of the sample is determined according to the following equation [56]:

9 $$\eta _{{{\text{PT}}}} = \frac{{hAT_{{{\text{max}}}} }}{I}$$

where *h*, *A*, *ΔT*_max_, and *I* were the heat transfer coefficient, surface area of the system, the temperature difference between the maximum temperature of the sample and ambient temperature, the light power, respectively. In order to get *hA*, *t* and *θ* defined as the ratio of Δ*T* to Δ*T*_max_ were introduced. The value of *hA* was derived according to the equation:

10 $$t = \frac{{\mathop \sum \nolimits_{i} m_{i} c_{{p,i}} }}{{hA}}\ln \theta$$

where *m* and *c*_p_ was the weight of coating and the specific heat of sample, which was determined using a method of sapphire with DSC instrument. Hence, *hA* can be acquired by calculating the aforementioned linear equation from the cooling period.

## Results and Discussion

The Ti_3_C_2_T_x_/CNTs/Co nanocomposites were synthesized as shown in Scheme 1. The Ti_3_C_2_T_x_ MXene sheets were prepared by ultrasonic delamination of Ti_3_C_2_T_x_-deionized water solution under the protection of Ar atmosphere, which was obtained by etching Ti_3_AlC_2_ particles with LiF and HCl solution. The sphere Co-MOF precursor was successfully fabricated by a facile microwave-assisted method. The sea urchin-like CNTs/Co nanocomposites were prepared by pyrolysis of Co-MOFs template under a reducing atmosphere, in which Co^2+^ ions were in situ reduced to 0D Co nanoparticles, and the organic components tended to be carbonized into 1D CNTs. Using an electrostatic assembly mechanism, the sea urchin-like CNTs/Co nanocomposites were adhered on the 2D Ti_3_C_2_T_x_ MXene sheets to form laminated Ti_3_C_2_T_x_/CNTs/Co nanocomposites.

**Fig. 1:**
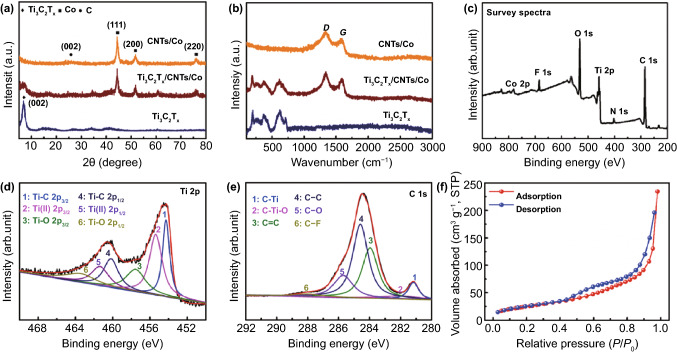
XRD curves (**a**) and Raman spectra (**b**) of Ti_3_C_2_T_x_, Ti_3_C_2_T_x_/CNTs/Co, and CNTs/Co nanocomposites. XPS survey spectra (**c**), Ti 2p XPS spectrum (**d**), C 1 s XPS spectrum (**e**), and N_2_ adsorption–desorption isotherms (**f**) of Ti_3_C_2_T_x_/CNTs/Co nanocomposites

The crystallographic structure and phase composition of the resultant nanocomposites were explored, as shown in Fig. 1a. The XRD curve of Ti_3_AlC_2_ (MAX) powder is illustrated in Figure S1, which was identified to (002), (104), and (105) planes of Ti_3_AlC_2_ with a hexagonal structure (JCPDS No. 52–0875). Figure 1a demonstrates the XRD pattern of Ti_3_AlC_2_ powder after the etching, delamination, and freeze-drying treatment. A distinguished diffraction peak at 2*θ* = 6.8° with an interlayer spacing of 1.30 nm corresponded to the (002) plane of the Ti_3_C_2_T_x_ sheets. The broadened diffraction peak of the Ti_3_C_2_T_x_ sample was attributed to the water and/or cationic intercalation in the hydrophilic and negatively charged Ti_3_C_2_T_x_ sheets [49]. In order to explore the thermal decomposition of Co-MOFs precursor, the thermal gravimetric behavior was analyzed, as shown in Fig. S2. It was seen that the weight loss reached up to 70.3 wt% and kept constant as the temperature increased above 575 ℃, which was corresponded to the thermal decomposition of Co-MOFs precursor. Thus, the pyrolysis temperature of 600 ℃ was selected in this work. After pyrolysis, for the formation of CNTs/Co nanocomposites, three diffraction peaks (44.2°, 51.5°, and 75.9°) were corresponded to ((111), (200), and (220)) planes of Co with cubic structure (JCPDS No. 89–7093). In addition, another diffraction peak at 2*θ* = 26.5° was assigned to the (002) plane of the hexagonal carbon (JCPDS No. 89–7213). Moreover, the XRD patterns of Ti_3_C_2_T_x_/CNTs/Co nanocomposites with different CNTs/Co content ratios (0, 25, 50, 75, and 100 wt%) are demonstrated in Fig. S3. For Ti_3_C_2_T_x_/CNTs/Co nanocomposites, the characteristic diffraction peaks were also well indexed with the Ti_3_C_2_T_x_ phase (6.8°), Co phase (44.2°, 51.5°, and 75.9°), and C phase (26.5°).

Raman spectra of Ti_3_C_2_T_x_, Ti_3_C_2_T_x_/CNTs/Co, and CNTs/Co nanocomposites are shown in Fig. 1b. For the Raman shift range from 100 to 800 cm^−1^, the Ti_3_C_2_T_x_, Ti_3_C_2_T_x_/CNTs/Co showed similar Raman spectra (Fig. S4). The modes at 195 and 718 cm^−1^ were assigned to the out-of-plane stretching vibration of Ti and C atoms, meanwhile, the modes (373 and 620 cm^−1^) corresponded to the in-plane modes of Ti, C, and surface functional group atoms (O, F, and OH) [57]. After the incorporation of CNTs/Co, two broad bands appeared at ~ 1330 and 1592 cm^−1^, which were assigned to the *D* band and *G* band, respectively [58]. Thus, it was confirmed that the carbon material was present in the hybrid. Generally, the *D* band indicates disorder and defects in carbon materials and the *G* band represents the graphitic degree with *sp*^2^ bonding [59, 60]. Moreover, the integrated intensity *I*_D_/*I*_G_ ratio of Ti_3_C_2_T_x_/CNTs/Co and CNTs/Co were 1.16 and 1.23, respectively. It would be due to the interaction between 2D Ti_3_C_2_T_x_ and CNTs/Co nanocomposites. Here, to further confirm the interaction between 2D Ti_3_C_2_T_x_ and CNTs/Co nanocomposites, X-ray photoelectron spectroscopy (XPS) was introduced to analyze the surface electronic states and chemical composition, as shown in Figs. 1c–e and S5. The XPS survey spectra demonstrated that the Ti_3_C_2_T_x_/CNTs/Co nanocomposites were composed of Ti, C, O, Co, N, and F (Fig. 1c). In special, there were two strong Co 2p and N 1 s peaks in the XPS survey spectra of Ti_3_C_2_T_x_/CNTs/Co nanocomposites, which were not found in the spectra of pure Ti_3_C_2_T_x_ sheets (Fig. S5a). The high-resolution XPS spectra of Ti 2p (Figs. 1d and S5b) was deconvolved into two peaks (Ti 2p_3/2_, Ti 2p_1/2_), in which the components centered at ~ 455.0, 455.9, and 458.1 eV were assigned to the Ti-C, Ti–O, and TiO_2_ bonds, respectively [25, 57, 61]. The high-resolution C 1 s (Fig. 1e) was fitted with six components located at ~ 281.4, 282.1, 284.8, 284.1, 286.2, and 288.0 eV, which corresponded to the C–Ti, Ti–C–O, C–C, C = C, C–O, and C–F bonds, respectively [62]. It was found that the peak intensity of the C–Ti bond in Ti_3_C_2_T_x_/CNTs/Co nanocomposites was smaller than that of Ti_3_C_2_T_x_ sheets (Fig. S5c), which was attributed to the contribution of CNTs/Co nanocomposites. Thus, it endowed Ti_3_C_2_T_x_ and CNTs/Co with strong electrostatic charges to induce the assembly of laminated Ti_3_C_2_T_x_/CNTs/Co nanocomposites. Moreover, the porous properties of Ti_3_C_2_T_x_/CNTs/Co nanocomposites were analyzed, as shown in Figs. 1f and S6. It was seen a long and narrow hysteresis loop, which was a typical IV-type isotherm. Thus, the Ti_3_C_2_T_x_/CNTs/Co nanocomposites exhibited mesoporous characteristics based on the IUPAC classification, which achieved a surface area of 93.57 m^2^ g^−1^ and a total pore volume of 0.36 cc g^−1^. The mesoporous structure might be derived from the rational construction of porous CNTs/Co nanoparticles and 2D Ti_3_C_2_T_x_ sheets. Based on the Maxwell–Garnett theory, the porous structure contributed to modulate permittivity values [63–65]. Additionally, porous composites showed the advantage of low density [66]. The room-temperature magnetic performance of all samples is shown in Fig. S7. Ti_3_C_2_T_x_ did not exhibit the magnetic hysteresis due to its lack of magnetic components. In contrast, Ti_3_C_2_T_x_/CNTs/Co had a saturation magnetization (*M*_s_) value of 32.7 emu g^−1^, remnant magnetization (*M*_r_) value of 8.1 emu g^−1^, and coercivity (*H*_c_) value of 368.5 Oe, which was lower than that of CNTs/Co (*M*_s_ of 57.1 emu/g, *M*_r_ of 14.5 emu g^−1^, and *H*_c_ of 401.2 Oe). The lower *M*_s_ value was caused by the non-magnetic composition of 2D Ti_3_C_2_T_x_ sheets and 1D CNTs, spin disorder and superparamagnetic relaxation of Co nanoparticles [67]. In addition, the lower coercivity value might be attributed to the interaction among 0D Co nanoparticles, porous carbon, and 2D Ti_3_C_2_T_x_ sheets and the limitation of the surface spin-canting effect [68].

**Fig. 2:**
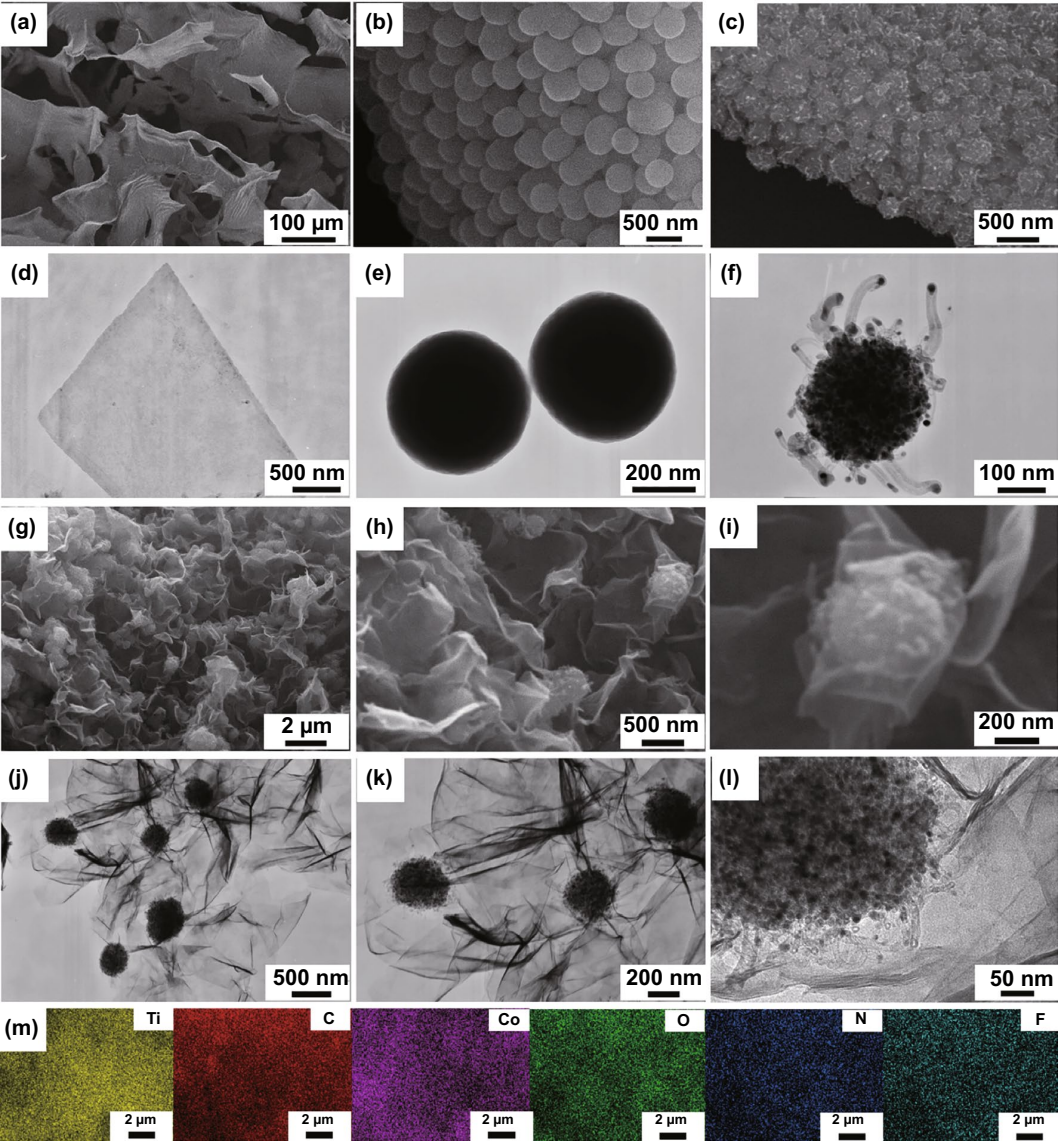
SEM images of **a** Ti_3_C_2_T_x_, **b** Co-MOFs, **c** CNTs/Co and **g–i** Ti_3_C_2_T_x_/CNTs/Co. TEM images of **d** Ti_3_C_2_T_x_, **e** Co-MOFs, **f** CNTs/Co, and **j-l** Ti_3_C_2_T_x_/CNTs/Co. **m** EDS mapping of Ti_3_C_2_T_x_/CNTs/Co

The morphology and microstructure of nanocomposites were characterized by SEM and TEM. Figure 2a shows that the Ti_3_C_2_T_x_ presented a sheet-like structure with an average size of 200 μm. The more energy-dispersive X-ray spectrometry (EDS) information is provided in Fig. S8. The TEM showed the flat flaky morphology of 2D Ti_3_C_2_T_x_ sheets (Fig. 2d) and the dark-field TEM image is illustrated in Fig. S9. Co-MOFs precursor had a smooth and uniformly spherical morphology with an average diameter of 300 nm (Fig. 2b, e). After calcination, a novel sea urchin-like structure was observed for CNTs/Co nanocomposites (Fig. 2c). Furthermore, the TEM image (Fig. 2f) illustrated that the CNTs/Co nanocomposites had a porous structure, in which 0D Co nanoparticles were well-dispersed inside the carbon spheres. Interestingly, the sphere was suspended with the extended and highly flexible 1D CNTs with 0D Co nanoparticles isolated inside. The elemental mapping further demonstrated that the Co, C, N, and O elements were evenly dispersed in the CNTs/Co nanocomposites (Fig. S10). Hence, the spherical Co-MOFs precursor can provide an ideal template for the preparation of novel sea urchin-like CNTs/Co nanocomposites. As for Ti_3_C_2_T_x_/CNTs/Co nanocomposites, the CNTs/Co nanocomposites were anchored onto the surface of the 2D Ti_3_C_2_T_x_ sheets (Fig. 2g–i), which was well consistent with the TEM results of Ti_3_C_2_T_x_/CNTs/Co nanocomposites (Fig. 2j–l). The EDS mapping of the SEM image in Fig. 2g further demonstrated that the Ti, C, Co, O, N and F elements were located in the Ti_3_C_2_T_x_/CNTs/Co nanocomposites (Fig. 2m). Thus, SEM, TEM, and EDS jointly confirmed the successful construction of laminated Ti_3_C_2_T_x_/ CNTs/Co nanocomposites.

**Fig. 3:**
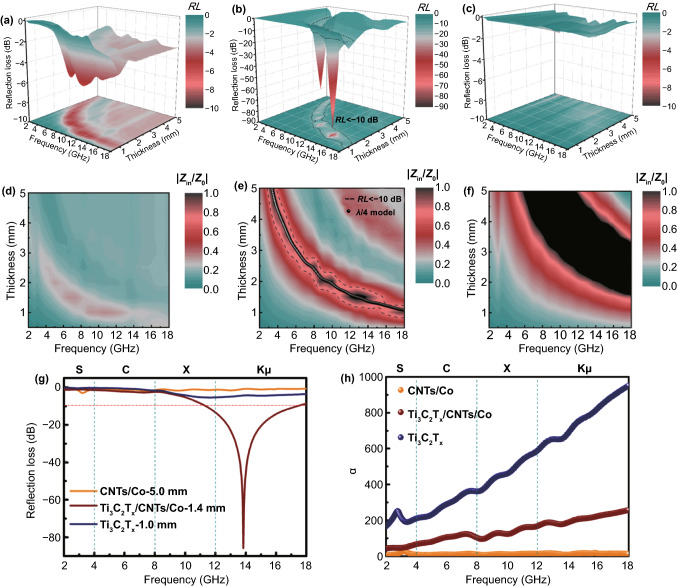
3D representations of *RL* values for (**a**) Ti_3_C_2_T_x_, (**b**) Ti_3_C_2_T_x_/CNTs/Co, and (**c**) CNTs/Co. 2D contour map representation of modulus of relative input impedance (|*Z*_in_/*Z*_0_|) for (**d**) Ti_3_C_2_T_x_, (**e**) Ti_3_C_2_T_x_/CNTs/Co, and (**f**) CNTs/Co. (**g**) The *RL* curves of CNTs/Co-5.0 mm, Ti_3_C_2_T_x_/CNTs/Co-1.4 mm, and Ti_3_C_2_T_x_-1.0 mm. (**h**) The attenuation constant (*α*) of Ti_3_C_2_T_x_, Ti_3_C_2_T_x_/CNTs/Co, and CNTs/Co nanocomposites

The reasonable design and development endowed the Ti_3_C_2_T_x_/CNTs/Co nanocomposite with excellent electromagnetic absorbing performances. Figure 3 shows the 3D representations of frequency and thickness-dependent reflection loss (*RL*) values for the resultant nanocomposites. The absorption peak shifted to the high-frequency band with decreasing the matching thickness, which can be explained by the quarter-wavelength (*λ*/4) matching model ($t_{m} = n\lambda /4 = nc/\left( {4f_{m} \sqrt {\left| {_{r} } \right|\left| {_{r} } \right|} } \right)$) [69–71]. When matching thickness (*t*_m_) and matching frequency (*f*_m_) satisfied this model, the reflected electromagnetic wave at the absorber-air interface was canceled out via destructive interference, thereby achieving the attenuation of the electromagnetic wave energy, which was due to the 180° phase difference between the incident and reflected electromagnetic wave in the absorbent [72–74]. Apparently, the Ti_3_C_2_T_x_/CNTs/Co nanocomposite conformed to this model, as shown in Fig. S11. Here, the minimum reflection loss (*RL*_min_), effective absorbing bandwidth (EAB, *RL* < −10 dB), and thickness (*d*) were introduced to evaluate the electromagnetic wave absorption performances. When the *RL* value is less than −10 dB, 90% of the incident wave can be absorbed [75]. As shown in Fig. 3a, c, and g, the *RL* values for Ti_3_C_2_T_x_ and CNTs/Co were above −10 dB in the thickness range of 0.5–5.0 mm, which demonstrated that the Ti_3_C_2_T_x_ and CNTs/Co were not suitable for practical applications of microwave absorption. In contrast, for Ti_3_C_2_T_x_/CNTs/Co, the EAB was 14.1 GHz covering from 2.9 to 18.0 GHz range in the thickness range of 1.0–5.0 mm. As shown in Fig. 3g, the RL_min_ of −85.8 dB at the frequency of 13.8 GHz with an ultrathin thickness of 1.4 mm was achieved for Ti_3_C_2_T_x_/CNTs/Co nanocomposites with 50 wt% content of CNTs/Co. In addition, in order to explore the influence of CNTs/Co content on the microwave absorbing performances, the microwave absorption of Ti_3_C_2_T_x_/CNTs/Co nanocomposites with two extra content of CNTs/Co (25 and 75 wt%) was studied and is shown in Fig. S12. The RL_min_, EAB, and *d* changed from −20 dB, 3.0 GHz, and 2.0 mm for Ti_3_C_2_T_x_/CNTs/Co _(25 wt%)_, to −12 dB, 1.5 GHz, and 1.0 mm for Ti_3_C_2_T_x_/CNTs/Co _(75 wt%)_. Here, the electromagnetic wave absorption performance of the MXene-based composites in recent years is listed in Table S1. Thus, it was indicated that the Ti_3_C_2_T_x_/CNTs/Co exhibited the enhanced electromagnetic wave absorption via rationally constructing 2D Ti_3_C_2_T_x_, 1D CNTs, and 0D Co nanocomposites. Considering the outstanding electromagnetic wave absorption, the hierarchical Ti_3_C_2_T_x_/CNTs/Co nanocomposites have promising application prospects in artificial intelligence electronic equipment and ongoing communication technology.

In order to reveal the enhanced mechanism of microwave absorption, the mechanism of conduction/dielectric/magnetic loss was thoroughly investigated. The frequency-dependent electromagnetic parameters (the complex permittivity (*ε'*, *ε*″) and the complex permeability (*μ'*, *μ*″)) were analyzed (Fig. S13). Among them, the *ε'* and *μ'* were related to the storage of electric and magnetic energy, while *ε*″ and *μ*″ were associated with the dissipation of electric and magnetic energy [76, 77]. The dielectric loss tangent (tan*δ*_e_ = *ε*″/*ε'*) and magnetic loss tangent (tan*δ*_m_ = *μ*″/*μ'*) were calculated to assess the dielectric loss and magnetic loss, respectively, as shown in Fig. S13. With increasing the frequency, the permittivity gradually decreased because of the frequency dispersion effect [78]. The CNTs/Co had relatively low electromagnetic parameters, which indicated that it was almost transparent to microwaves. It was found that the *ε*″ values gradually enlarged with the assembly with MXene and pure MXene obtained the largest *ε*″ values. According to the free electron theory $\left( {\varepsilon ^{\prime\prime} = \frac{{\varepsilon _{s} - \varepsilon _{\infty } }}{{1 + \left( {2\pi f} \right)^{2} \tau ^{2} }}2\pi f\tau + \frac{\sigma }{{2\pi f\varepsilon _{0} }}} \right)$ [32, 79, 80], a higher conductivity (*σ*) will contribute to a higher *ε*″ value, thereby strengthening the ability to dissipate electric energy [81]. For the hierarchical Ti_3_C_2_T_x_/CNTs/Co nanocomposites, the Ti_3_C_2_T_x_ with a high conductivity provided a three-dimensional (3D) conductive network, which leading to the increase in *ε*″ values and contributing to the conductive loss [80]. Given the 3D microstructure built with 1D CNTs and 2D MXene, the conduction loss can play a fundamental role in the microwave attenuation. Here, two electronic transport modes were proposed, i.e., migrating electrons and hopping electrons [80]. In response to the interaction of the incident electromagnetic wave with the hierarchical Ti_3_C_2_T_x_/CNTs/Co nanocomposites, the migrating electrons can transport in the 2D MXene and 1D CNTs, while the hopping electrons would jump across the defects of the MXene, or jump to the CNTs [5, 80], so as to form a dense micro-current network. In addition, several resonant peaks of the *ε*″–*f* and tan*δ*_e_–*f* curves are observed in Fig. S13, which indicating the contribution of the dielectric relaxation. The *ε*′−*ε*″ relation $\left( {\varepsilon ^{'} - \frac{{\varepsilon _{s} + \varepsilon _{{\infty )}} }}{2})^{2} + \left( {\varepsilon ^{\prime\prime}} \right)^{2} = \left( {\frac{{\varepsilon _{s} - \varepsilon _{\infty } }}{2}} \right)^{2} } \right)$ was provided by the Debye theory and the free electron theory [82, 83]. Several semicircles were observed in all *ε*′−*ε*″ curves at the high frequency (Fig. S14). A Cole–Cole semicircle was associated with one relaxation process [84, 85]. The charge accumulation at the interface and the formation of the dipole will form different semicircles [5]. It revealed that the hierarchical Ti_3_C_2_T_x_/CNTs/Co nanocomposites had multiple dipolar relaxation processes under an alternating electromagnetic field [86]. Moreover, the linear relationship of low-frequency *ε*′−*ε*″ curves was related to the conduction loss [87]. For polarization, the abundant heterogeneous interfaces including MXene/CNTs, CNTs/Co nanoparticles, and layered MXene interfaces produced a large number of interfacial polarization [88, 89]. Furthermore, as the dipoles, numerous defects and polar functional groups with different electronegativity would induce the dipole polarization [90]. Thus, the assembly of hierarchical Ti_3_C_2_T_x_/CNTs/Co nanocomposites with 2D MXene, CNTs/Co, and porous carbon would enhance the active interfaces and local polarization, which contributed to the dielectric loss. Given the magnetic behavior of 0D Co nanoparticles, the magnetic loss was important for strengthening electromagnetic wave dissipation. Several resonance peaks of the permeability values in the composites are observed in Fig. S13. Generally, permeability value was related to the anisotropy constant (*K*_1_), saturation magnetization (*M*_s_), and grain size (*D*) according to the Globus equation *μ* ∝ *M*_s_^2^*D*/*K*_1_ [91]. Here, the hysteresis, domain wall resonance, eddy-current loss, and ferromagnetic resonance of the magnetic loss mechanism were discussed [92]. The hysteresis loss can be negligible in the weak applied field. The frequency of domain wall resonance loss is mainly in the frequency range of megahertz. The eddy-current loss effect was studied by the frequency-dependent *μ*″(*μ*′)^−2^*f*^−1^ curves [93–95]. The variation of *μ*″(*μ*′)^−2^*f*^−1^ value was observed (Fig. S15), suggesting that the eddy-current loss hardly worked in this frequency band. Due to the confinement effect and the small size effect of the 0D Co nanoparticles [96, 97], the ferromagnetic resonance mainly caused the magnetic loss of the hierarchical Ti_3_C_2_T_x_/CNTs/Co nanocomposites, where the natural resonance worked at the low frequency (< 10 GHz) and exchange resonance reacted in the high frequency (> 10 GHz) [24].

In order to further understand the underlying mechanism of enhanced electromagnetic wave absorption, the capture ability and attenuation capability of the incident microwave were further discussed. In general, impedance matching is a key factor to ensure more capture of the incident microwave and reduce the surface reflection of the absorber, which can be evaluated by the modulus of relative input impedance (|*Z*_in_/*Z*_0_|). Good impedance matching requires that the |*Z*_in_/*Z*_0_| is equal or close to 1.0. Figure 3d–f) shows 2D contour map representations of the thickness and frequency-dependent |*Z*_in_/*Z*_0_| values. The |*Z*_in_/*Z*_0_| value was much below 1 for Ti_3_C_2_T_x_ and the |*Z*_in_/*Z*_0_| value was much above 1 for CNTs/Co, which led to a large amount of reflection of microwave and poor microwave absorption. However, Ti_3_C_2_T_x_/CNTs/Co obtained a superior impedance matching with a larger area of the |*Z*_in_/*Z*_0_| value which is close to 1, ensuring that most of the incident microwave were captured and entered the inside of the absorber. The impedance matching was associated with electromagnetic parameters, which was originated from the component and microstructure of the composites. Here, the modulated permittivity was related to the conductivity and novel architecture while the permeability was derived from ferromagnetic Co nanoparticles, which contributed to the superior impedance matching in laminated Ti_3_C_2_T_x_/CNTs/Co nanocomposites. Furthermore, the microwave attenuation efficiency was evaluated by the attenuation constant *α* [98–100]. It was found that the microwave attenuation capability was determined by the dielectric loss and magnetic loss capability. Figure 3h demonstrates the frequency-dependent attenuation constant *α*. The order of *α* value was CNTs/Co < Ti_3_C_2_T_x_/CNTs/Co < Ti_3_C_2_T_x_, which corresponding to the microwave attenuation ability. Although the Ti_3_C_2_T_x_ has the greatest dissipation capability, it still showed unsatisfactory microwave absorption performances. Here, the contribution of impedance matching and dissipation capacity was further investigated. Frequency-dependent |*Z*_in_/*Z*_0_|, *α*, and *RL* values are shown in Fig. S16. *RL* value reached the minimum value when the |*Z*_in_/*Z*_0_| value was approaching 1 with the moderate *α* value (42–257). However, the |*Z*_in_/*Z*_0_| of CNTs/Co was far above 1 with a low *α* value (5–27), while the |*Z*_in_/*Z*_0_| value of Ti_3_C_2_T_x_ was much below 1 with the largest *α* value (165–951). It would significantly reflect an abundant incident microwave and eventually lead to poor microwave absorption. Thus, the combination of attenuation ability and promoted impedance matching contributed to outstanding microwave absorbing performances in the laminated Ti_3_C_2_T_x_/CNTs/Co nanocomposites.

**Fig. 4:**
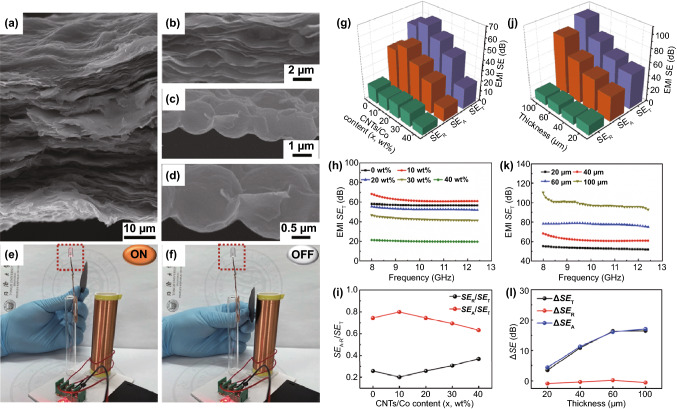
Cross-sectional SEM image of Ti_3_C_2_T_x_/CNTs/Co film (**a–d**). The photograph of wireless power-transfer circuit without Ti_3_C_2_T_x_/CNTs/Co film (**e**), LED on, and with Ti_3_C_2_T_x_/CNTs/Co film (**f**), LED off. (**g**) 3D histogram of average *SE*_T_, *SE*_A_, and *SE*_R_ value, (**h**) average *SE*_T_, and (**i**) *SE*_A_/*SE*_T_ and *SE*_R_/*SE*_T_ ratio of 40-μm-thick Ti_3_C_2_T_x_/CNTs/Co nanocomposites with different contents of CNTs/Co. (**j**) 3D histogram of average *SE*_T_, *SE*_A_, and *SE*_R_ value, (**k**) average *SE*_T_ of Ti_3_C_2_T_x_/CNTs/Co_(10 wt%)_ nanocomposites with different thicknesses, and (**l**) comparison of EMI Δ*SE*_T_, Δ*SE*_A_, and Δ*SE*_R_ value versus thickness of Ti_3_C_2_T_x_/CNTs/Co_(10 wt%)_ and Ti_3_C_2_T_x_ films

Generally, the high electrical conductivity of the material is a prominent factor in achieving an excellent EMI shielding performance [101–103]. Here, the EMI shielding efficiency of Ti_3_C_2_T_x_/CNTs/Co nanocomposites was investigated in depth, considering the remarkable conductivity of the 2D Ti_3_C_2_T_x_ and 1D CNTs in the composites. In Fig. 4a, the layered Ti_3_C_2_T_x_/CNTs/Co film with slight undulating structures was clearly identified. Figure 4b shows that the Ti_3_C_2_T_x_ MXene layer exhibited a fluffy stacked interconnected microstructure, in which the CNTs/Co nanoparticles were distributed inside, indicating that CNTs/Co nanoparticles connected between the Ti_3_C_2_T_x_ layers. The high-magnification SEM images (Fig. 4c, d) revealed that the Ti_3_C_2_T_x_ sheets were wrapped on the CNTs/Co nanoparticles. The EMI shielding properties of 40-um-thick Ti_3_C_2_T_x_/CNTs/Co film with different CNTs/Co contents were explored, as shown in Figs. 4g, h and S17. With increasing the CNTs/Co content, the shielding efficiency first increased and then decreased. The Ti_3_C_2_T_x_/CNTs/Co film with 10 wt% of CNTs/Co showed the highest *SE*_T_ value of 62.0 dB. Figure 4i provides the *SE*_R_/*SE*_T_ and *SE*_A_/*SE*_T_ value versus CNTs/Co content. The *SE*_A_/*SE*_T_ value was much larger than that of *SE*_R_/*SE*_T_, indicating that the *SE*_A_ made a more contribution to the *SE*_T_. Interestingly, the *SE*_A_/*SE*_T_ reached the largest value, which corresponding to the excellent shielding performance. In addition, the thickness-dependent EMI shielding efficiency of Ti_3_C_2_T_x_/CNTs/Co film with 10 wt% of CNTs/Co was also investigated and is demonstrated in Figs. 4j, k and S18. It was found that the total *SE*_T_ reached the maximum value of 110.1 dB with the thickness increasing to 100 μm. To compare the EMI *SE* of the Ti_3_C_2_T_x_/CNTs/Co film and the Ti_3_C_2_T_x_ film, the EMI Δ*SE*_T_, Δ*SE*_A_, and Δ*SE*_R_ values of the Ti_3_C_2_T_x_/CNTs/Co_(10 wt%)_ and Ti_3_C_2_T_x_ films with different thicknesses are analyzed in Figs. 4l and S18. With increasing the film thickness, there was no obvious change of Δ*SE*_R_ value between the Ti_3_C_2_T_x_/CNTs/Co_(10 wt%)_ and the Ti_3_C_2_T_x_ films, whereas the gradual improvement of Δ*SE*_A_ value was observed. Most importantly, the Δ*SE*_T_ value had a similar trend with the Δ*SE*_A_ value, which suggesting an absorption-dominated electromagnetic wave attenuation mechanism in the Ti_3_C_2_T_x_/CNTs/Co system. Furthermore, the EMI shielding efficiency of MXene-based composites is summarized in Table S2. It was shown that the Ti_3_C_2_T_x_/CNTs/Co films exhibited a favorable EMI shielding performance.

Here, we further analyzed the EMI shielding mechanisms of the Ti_3_C_2_T_x_/CNTs/Co film. According to Simon’s formula [9], the shielding efficiency was closely related to the conductivity and thickness of shielding materials. Figure S19 shows the electric conductivity of the Ti_3_C_2_T_x_/CNTs/Co films with different CNTs/Co contents and thicknesses. As the CNTs/Co content increased to 10 wt%, the electric conductivity increased to a maximum value of 3571 S cm^−1^, which was corresponded to the largest EMI shielding efficiency. Moreover, as the film thickness increased, the electric conductivity of the Ti_3_C_2_T_x_/CNTs/Co films gradually increased to the highest value of 5108 S cm^−1^, which was greater than that the electrical conductivity of the Ti_3_C_2_T_x_ films (3410 S cm^−1^). Hence, it contributed to the excellent EMI shielding performance in the Ti_3_C_2_T_x_/CNTs/Co films. Generally, EMI shielding performance was originated from the mobile charge carriers, electric/magnetic dipoles, and interior interfaces. Given the 3D architecture built with 0D Co nanoparticles, 1D CNTs, and 2D MXene, a 3D conductive network can efficiently consume electromagnetic energy. The electric polar functional groups as the dipoles induced the electric dipole polarization, while the ferromagnetic resonant of Co nanoparticles led to the magnetic polarization. In addition, a large number of heterogeneous interfaces in the Ti_3_C_2_T_x_/CNTs/Co films produced abundant interfacial polarization, which would further dissipate the electromagnetic energy. Moreover, the laminated porous structure caused the multiple reflections and scattering of the incident microwave, thereby enhancing energy dissipation. To verify the actual EMI shielding effect of Ti_3_C_2_T_x_/CNTs/Co films, a typical wireless power transmission system was built, as shown in Fig. 4e, f. The electromotive force generated by electromagnetic induction in the receiver coil can light up the light-emitting diode (LED) (Fig. 4e). When the Ti_3_C_2_T_x_/CNTs/Co film was inserted between the two coils, the LED light was turned off (Fig. 4f and Movie S1). It was due to the obstruction of electromagnetic transmission by the Ti_3_C_2_T_x_/CNTs/Co film. Thus, the obtained Ti_3_C_2_T_x_/CNTs/Co film was expected to achieve efficient EMI shielding in practical applications.

**Fig. 5:**
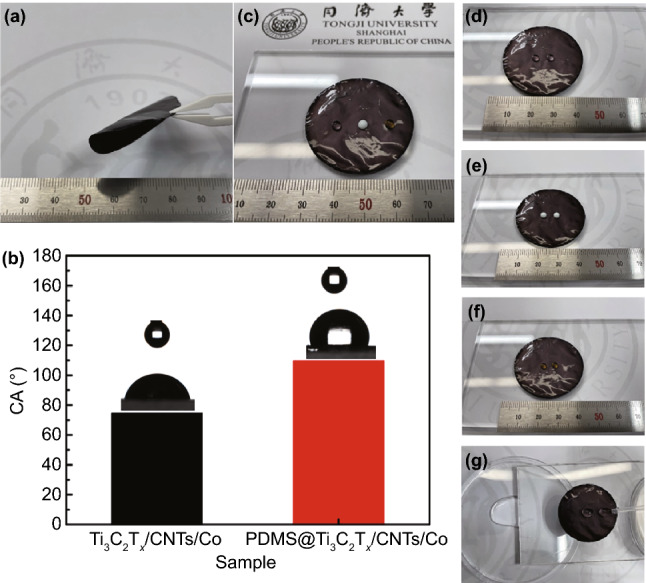
**a** Bending photograph of the PDMS@Ti_3_C_2_T_x_/CNTs/Co film. **b** Water contact angle (CA) of Ti_3_C_2_T_x_/CNTs/Co film and PDMS@Ti_3_C_2_T_x_/CNTs/Co film. Inset is a photograph of the measurement of water adhesion (3 μL water droplet) on the film surface. **c–f** Photograph of the liquid droplets of water (translucent), milk (white), and coffee (yellow) solution sitting on the surface, **g** optical photographs of self-cleaning test on coated filter paper. (Color figure online)

In view of the excellent electromagnetic wave absorption and EMI shielding efficiency of the Ti_3_C_2_T_x_/CNTs/Co nanocomposites, the water-resistant treatment of the surface is particularly important to maintain its electromagnetic attenuation performance in practical applications. In general, PDMS due to its transparency, hydrophobicity, high elasticity, biocompatibility, and easy modeling capability has been widely applied to electronic skins [104], sensors [105], and thermal management [106]. In this work, the Ti_3_C_2_T_x_/CNTs/Co film was coated by PDMS, which can provide the flexibility and hydrophobicity characters. Figure 5a illustrates the bending photograph of the PDMS@Ti_3_C_2_T_x_/CNTs/Co film, which confirmed that the PDMS@Ti_3_C_2_T_x_/CNTs/Co film had high flexibility. Furthermore, the water contact angle (CA) of the Ti_3_C_2_T_x_/CNTs/Co film and the PDMS@Ti_3_C_2_T_x_/CNTs/Co film was analyzed. Figure 5b shows that the CA value of the PDMS@Ti_3_C_2_T_x_/CNTs/Co film (110.3°) was larger than the CA value of the Ti_3_C_2_T_x_/CNTs/Co film (73.1°). Figure 5c–f shows that the liquid droplets (water, milk, and coffee) retained a spherical shape on the surface of the PDMS@Ti_3_C_2_T_x_/CNTs/Co film, exhibiting hydrophobicity. Figure 5g illustrates that the water droplets from a dropper rolled off the surface due to the hydrophobicity and negligible water adhesion to the PDMS@Ti_3_C_2_T_x_/CNTs/Co film. Therefore, the PDMS@Ti_3_C_2_T_x_/CNTs/Co film had remarkable flexibility and hydrophobicity characteristics, which would expand its practical application range in moist or wet environments.

**Fig. 6:**
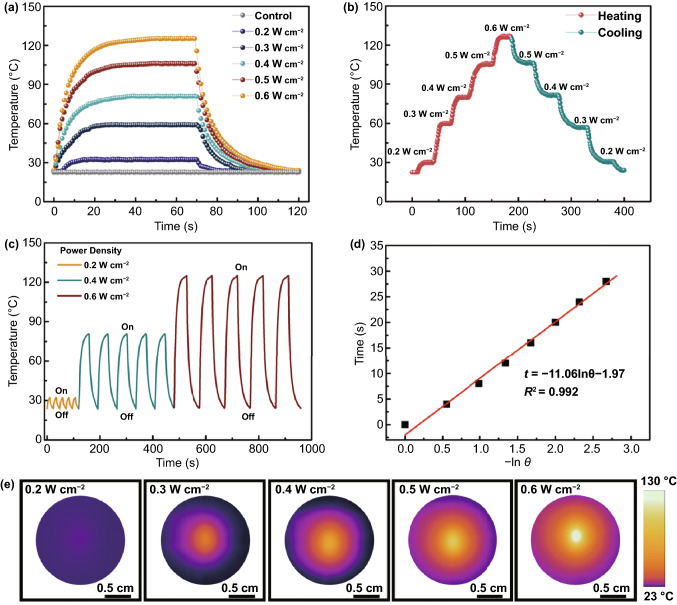
**a** Photothermal heating and cooling curves of the PDMS@Ti_3_C_2_T_x_/CNTs/Co film at different NIR laser power densities. **b** Tailored surface temperatures of PDMS@Ti_3_C_2_T_x_/CNTs/Co coating under gradually changing power density. **c** Heating curves of the PDMS@Ti_3_C_2_T_x_/CNTs/Co coating for five laser on/off cycles at different NIR laser power densities. **d** A linear fitting correlation between time (*t*) and -ln*θ* obtained from the cooling period. **e** Infrared thermographic photographs of the PDMS@Ti_3_C_2_T_x_/CNTs/Co coating under power densities rise from 0.2 to 0.6 W cm^−2^

In addition to the above-mentioned intriguing functions, the outstanding light-to-heat conversion performance endowed Ti_3_C_2_T_x_/CNTs/Co nanocomposites with potential application in wearable heaters for self-heating garments [107–110]. Here, the PDMS@Ti_3_C_2_T_x_/CNTs/Co film was exposed to an 808 nm NIR laser irradiation with different power densities (0.2, 0.3, 0.4, 0.5, and 0.6 W cm^−2^) to explore its photothermal performance. Figure 6a shows that the surface temperature of the PDMS@Ti_3_C_2_T_x_/CNTs/Co film reached about 33.5 ℃ within 20 s even under relatively low power density (0.2 W cm^−2^) irradiation. Overall, as the NIR light power densities increased, the surface temperature of the PDMS@Ti_3_C_2_T_x_/CNTs/Co film increased significantly and reached the maximum steady-state value. In particular, under exposure to continuous NIR light at the power density of 0.6 W cm^−2^, the surface temperature reaches a maximum value of 126.6 ℃ (Movie S2). It is very interesting that regardless of the light power density, a very fast thermal response time was observed during the heating process, which demonstrated a fast thermal response of the PDMS@Ti_3_C_2_T_x_/CNTs/Co film. In contrast, the PDMS film as a control group had no temperature variation under the same irradiation process. It was indicated that the PDMS@Ti_3_C_2_T_x_/CNTs/Co film exhibited an efficient photothermal conversion. Figure 6b reveals the excellent real-time temperature dependence on the light power density under the increasing power density stepwise from 0.2 to 0.6 W cm^−2^, and decreasing to 0.2 W cm^−2^. It demonstrated a controllable light-to-heat performance of the PDMS@Ti_3_C_2_T_x_/CNTs/Co film. Figure 6e shows infrared thermographic photographs of the PDMS@Ti_3_C_2_T_x_/CNTs/Co film with the power density changing from 0.2 to 0.6 W cm^−2^. Clearly, the thermal image showed a uniform temperature distribution, which had high application prospects in photothermal heaters.

In order to further explore the photothermal stability and recyclability of the PDMS@Ti_3_C_2_T_x_/CNTs/Co film, the recycling temperature change was assessed under different NIR irradiation (0.2, 0.4, and 0.6 W cm^−2^) and then naturally cooled to the ambient temperature for five on/off light cycles. Figure 6c shows the stable and regular ascending and descending temperature cycles corresponding to turning on and off the light during the whole cycling process, demonstrating outstanding photothermal stability and recyclability of the PDMS@Ti_3_C_2_T_x_/CNTs/Co film. The photothermal conversion efficiency is one of the important factors to evaluate heating performance. Figure 6d presents a linear fitting correlation between time (*t*) and ln*θ* taking a light power density of 0.6 W cm^−2^ as an example. The photothermal conversion efficiency of the PDMS@Ti_3_C_2_T_x_/CNTs/Co film was as high as 29.5%, which was higher than that of SnS nanosheets (24%) [111] and MoS_2_ (24.37%) [112], and slightly smaller than that of PDMS@m-Ti_3_C_2_T_x_/d-Ti_3_C_2_T_x_ coating (30.3%) [109]. Here, the photothermal conversion mechanism was explored in depth. The material composition was of importance role in absorbing photons and converting the photon energy to heat. Hence, the synergy of the unique localized surface plasmon resonance (LSPR) effect in 0D Co nanoparticles [113], the conjugation and hyperconjugation effect in 1D CNTs [114] as well as the strong light absorption and the LSPR effect in 2D Ti_3_C_2_T_x_ MXenes [115] endowed the PDMS@Ti_3_C_2_T_x_/CNTs/Co film with the favorable photothermal conversion performance. In addition, by rationally designing the microstructure to harvest light to the greatest extent, and adopting a suitable thermal insulation layer to minimize heat loss, the photothermal performance can be further significantly improved [107]. Attributing to the multiple composition and novel structures, the PDMS@Ti_3_C_2_T_x_/CNTs/Co film enabled an outstanding photothermal performance. Therefore, the excellent photothermal performance with excellent cycle stability and adjustability can further broaden the possible application range of this novel multifunctional material.

## Conclusion

We demonstrated the 2D/1D/0D construction of Ti_3_C_2_T_x_/CNTs/Co nanocomposites with excellent electromagnetic wave absorption, EMI shielding efficiency, flexibility, hydrophobicity, and photothermal functions via a facile method of microwave-assisted, in situ carbonization and electrostatic assembly process. The sea urchin-like CNTs/Co nanocomposites were introduced on 2D Ti_3_C_2_T_x_ MXene sheets to form laminated Ti_3_C_2_T_x_/CNTs/Co nanocomposites to improve the electromagnetic wave absorption and enhance the EMI shielding efficiency. As expected, a strong reflection loss of −85.8 dB, a broad EAB of 6.1 GHz, an ultrathin thickness of 1.4 mm, and an ultralow filler loading of 5 wt% were achieved for the laminated Ti_3_C_2_T_x_/CNTs/Co nanocomposites by the improved attenuation capability and optimized impedance matching. The investigation of the underlying mechanism revealed that the electromagnetic wave absorbing performances were enhanced by the synergistic effects between the conduction loss originated from the electronic transport in the conductive network of 1D CNTs and 2D Ti_3_C_2_T_x_, the dielectric loss stemmed from the dipole polarization and abundant interface in the laminated structure, and the magnetic loss derived from the ferromagnetic resonance of 0D Co nanoparticles. The Ti_3_C_2_T_x_/CNTs/Co film exhibited a high EMI *SE* of 110.1 dB, which originated from the excellent electrical conductivity, electric and or magnetic dipole polarization, interfacial polarization, natural resonance, and multiple internal reflections. Moreover, PDMS rendered the hydrophilic hierarchical Ti_3_C_2_T_x_/CNTs/Co hydrophobic with a water contact angle of ~ 110.3°, which can prevent the degradation/oxidation of the MXene under high humidity conditions. Interestingly, the PDMS@Ti_3_C_2_T_x_/CNTs/Co film exhibited an excellent photothermal conversion performance with high thermal cycle stability and tenability. Thus, our multifunctional Ti_3_C_2_T_x_/CNTs/Co nanocomposites possessed a unique blend of outstanding electromagnetic wave absorption and EMI shielding efficiency, light-driven heating performance, flexibility, and water-resistant features, which was highly promising for the next generation of intelligent electromagnetic attenuation systems.

## Supplementary Information

Below is the link to the electronic supplementary material.

Supplementary file1 (PDF 1871 KB)

Supplementary file2 (MP4 2755 KB)

Supplementary file3 (MP4 11425 KB)
