# Supplementary material for: Flexible and Waterproof 2D/1D/0D Construction of MXene-Based Nanocomposites for Electromagnetic Wave Absorption, EMI Shielding, and Photothermal Conversion
Source: Nanomicro Lett. 2021 Jun 25;13:150. doi: 10.1007/s40820-021-00673-9 (PMC8233447; doi:10.1007/s40820-021-00673-9)
Supplement: Supplementary file 1 — Supplementary file1 (PDF 1871 KB) [file 40820_2021_673_MOESM1_ESM.pdf]

Supporting Information for

Flexible and Waterproof 2D/1D/0D Construction of MXene-Based Nanocomposites for Electromagnetic Wave Absorption, EMI Shielding and Photothermal Conversion

Zhen Xiang[1], Yuyang Shi[1], Xiaojie Zhu, Lei Cai[1], and Wei Lu[1,] *

[1]Shanghai Key Lab of D&A for Metal-Functional Materials, School of Materials Science & Engineering, Tongji University, Shanghai 201804, P. R. China

*Corresponding author. E-mail: weilu@tongji.edu.cn (Wei Lu)

Supplementary Tables and Figures

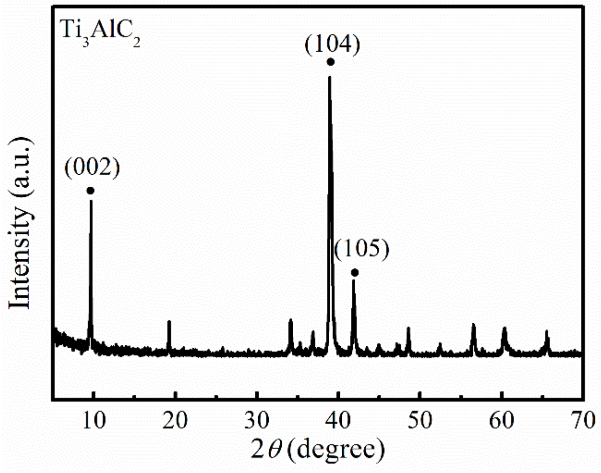

Fig. S1 XRD curve of Ti_3AlC_2 powder

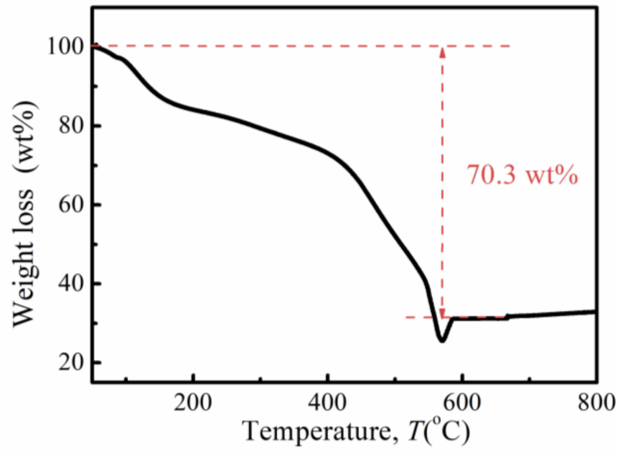

Fig. S2 TG curve of Co-MOFs precursor

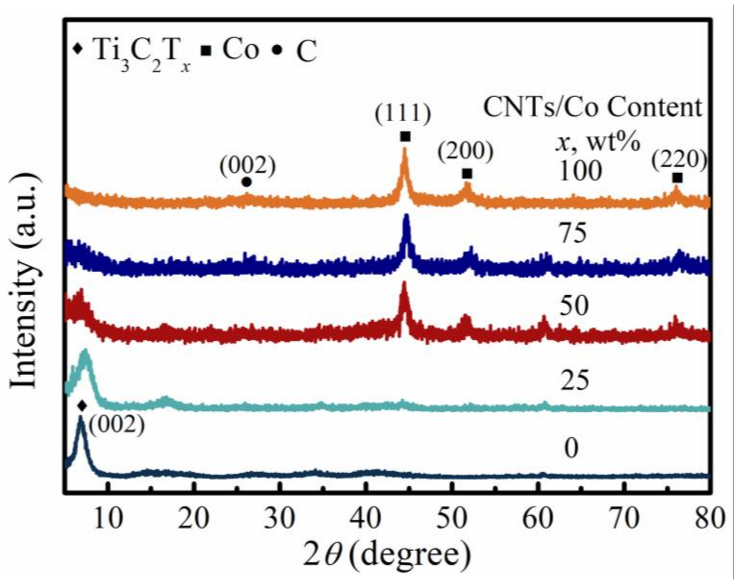

Fig. S3 XRD patterns of $Ti_3C_2T_x$/CNTs/Co nanocomposites with different CNTs/Co ratios (0, 25 wt%, 50 wt%, 75 wt%, 100 wt%)

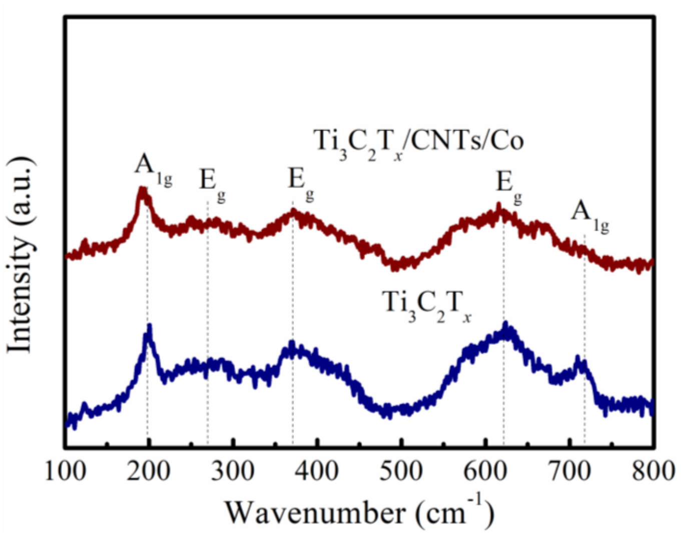

Fig. S4 Raman spectra of $Ti_3C_2T_x$ and $Ti_3C_2T_x$/CNTs/Co in the wavenumber of 100-800 cm^{-1}

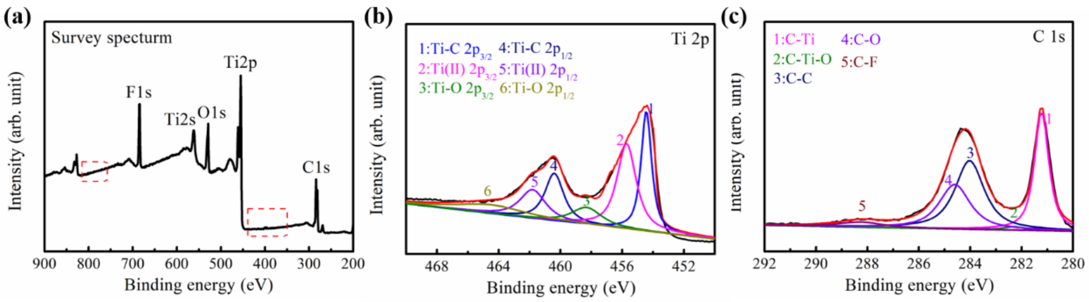

Fig. S5 XPS survey spectra (**a**), Ti 2p XPS spectrum (**b**), and C 1s XPS spectrum (**c**) of $Ti_3C_2T_x$ sheets

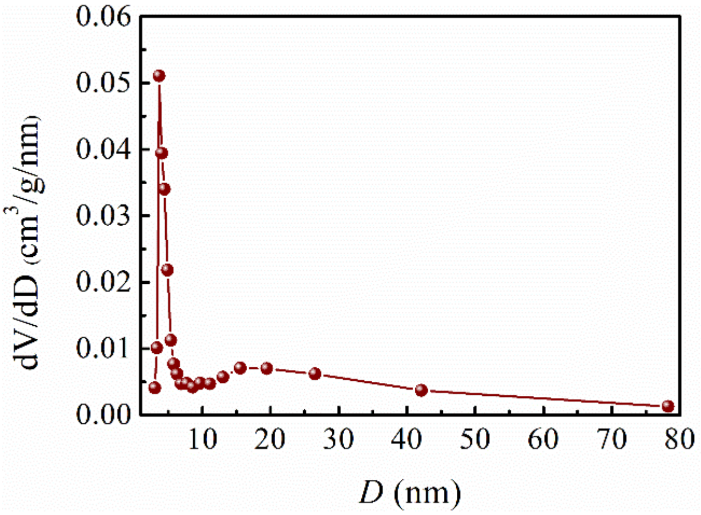

Fig. S6 Pore size distribution of $Ti_3C_2T_x$/CNTs/Co nanocomposites

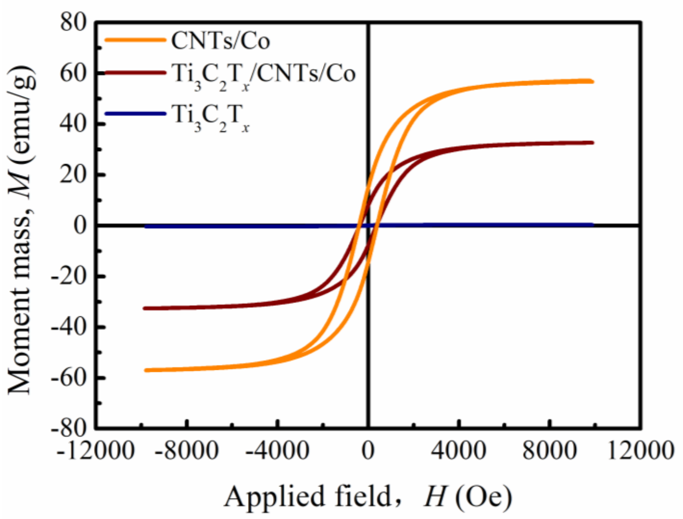

Fig. S7 Room temperature magnetic hysteresis loops of CNTs/Co, $Ti_3C_2T_x$/CNTs/Co, and $Ti_3C_2T_x$

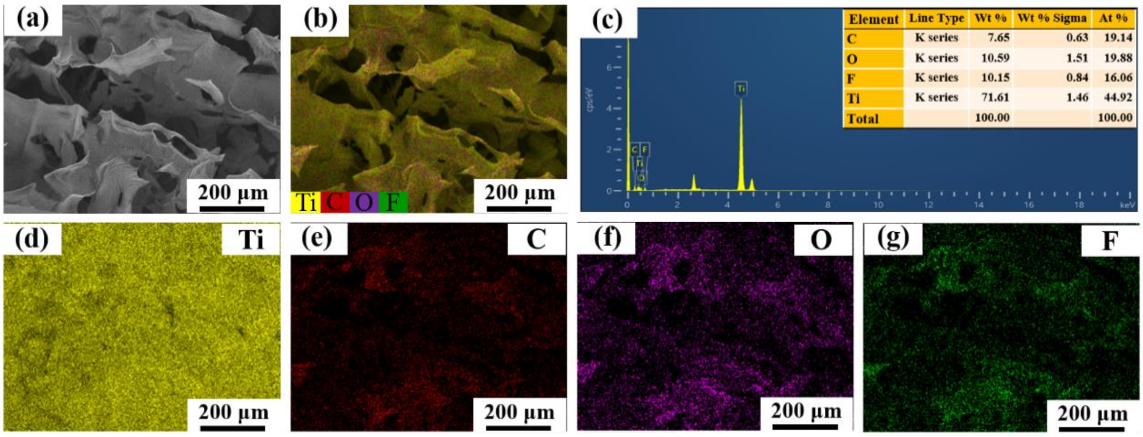

Fig. S8 SEM and EDS images of $Ti_3C_2T_x$ (T=O, F) MXene sheets

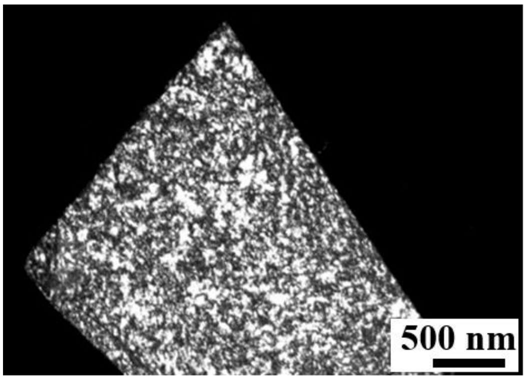

Fig. S9 Darkfield TEM image of $Ti_3C_2T_x$ sheet

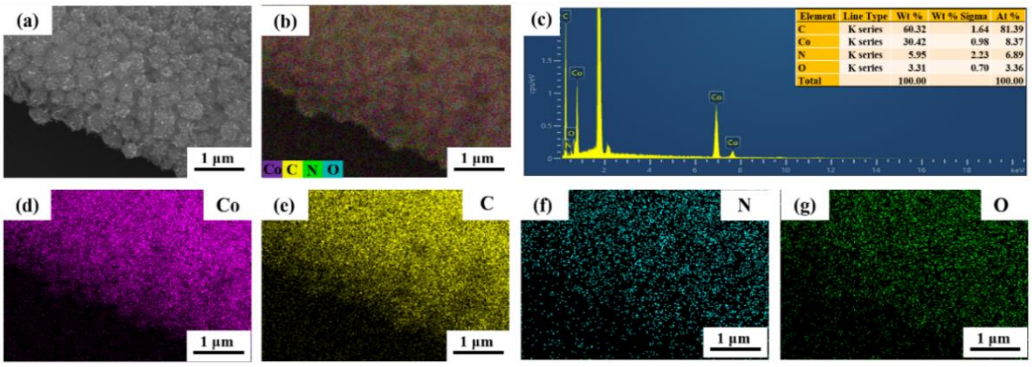

Fig. S10 SEM and EDS images of CNTs/Co nanocomposites

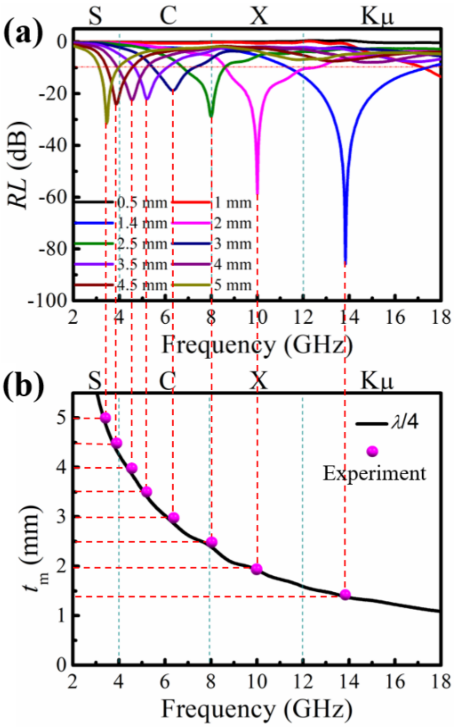

Fig. S11 (**a**) *RL* value versus frequency and thicknesses, (**b**) Relationship between simulated matching thickness t_m and peak frequency of $Ti_3C_2T_x$/CNTs/Co nanocomposites

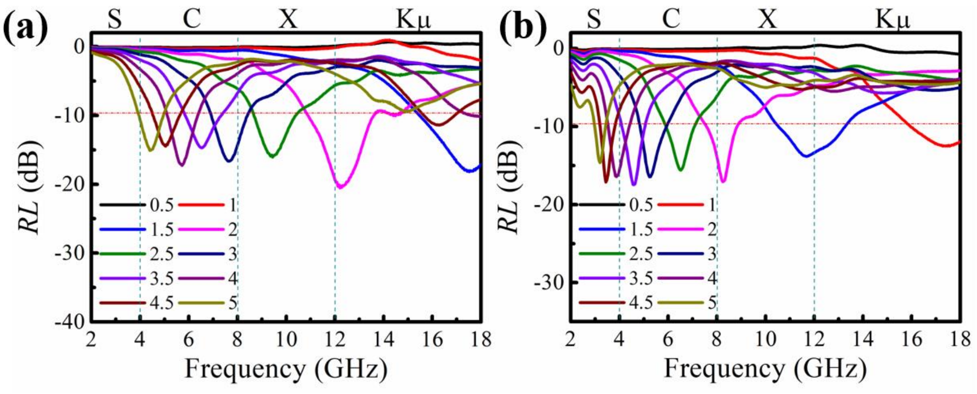

Fig. S12 *RL* curves of $Ti_3C_2T_x$/CNTs/Co nanocomposites with 25 wt% (**a**) and 75 wt% (**b**) content of CNTs/Co

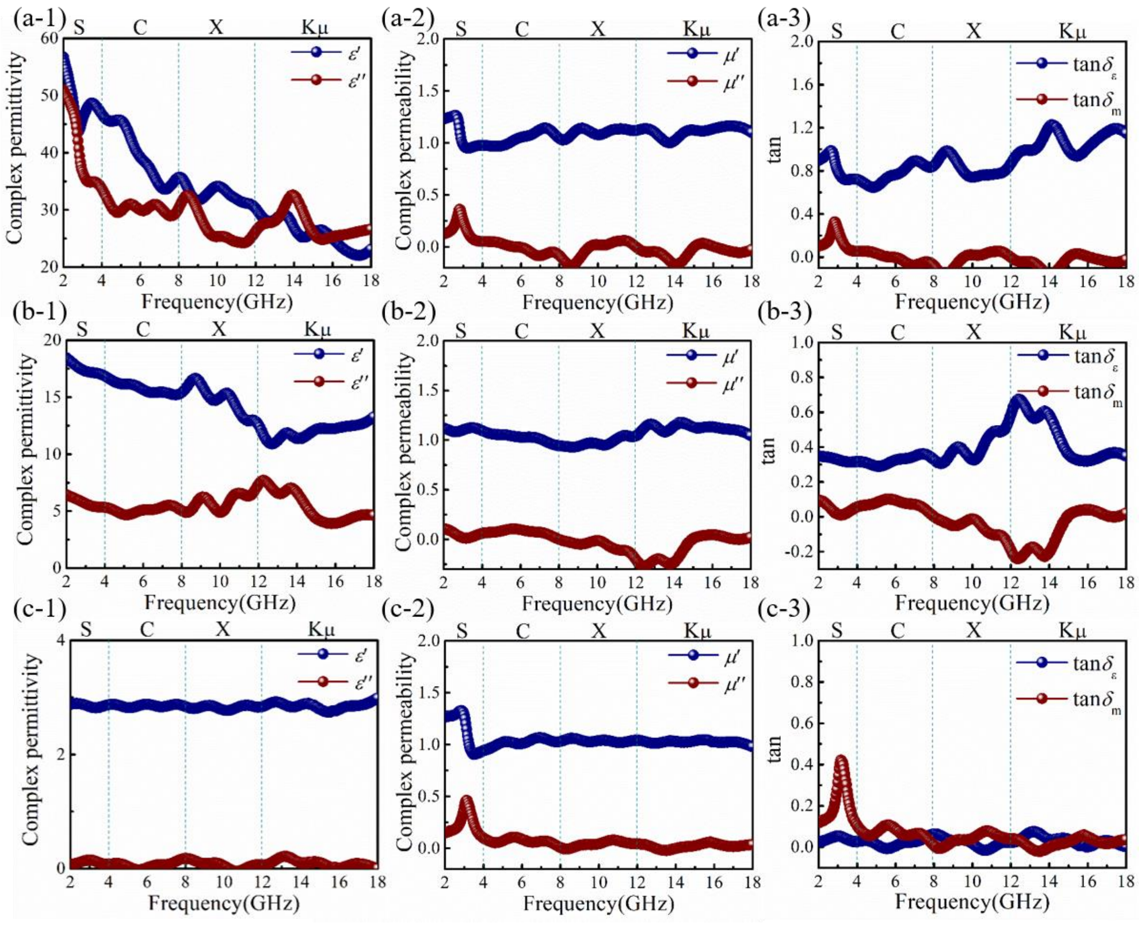

Fig. S13 Frequency dependence of permittivity (ε', ε''), permeability (μ', μ'') and loss tangent (tanδ_m, tanδ_e) of $Ti_3C_2T_x$ (**a-1, a-2, a-3**), $Ti_3C_2T_x$/CNTs/Co (**b-1, b-2, b-3**), and CNTs/Co (**c-1, c-2, c-3**)

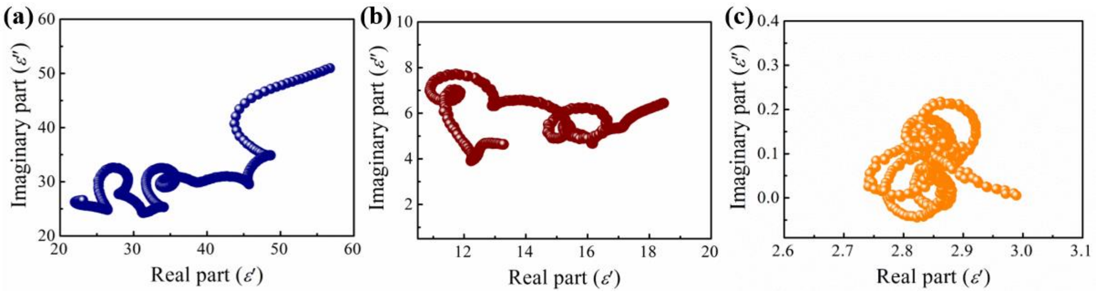

Fig. S14 ε'-ε'' curves of $Ti_3C_2T_x$ (**a**), $Ti_3C_2T_x$/CNTs/Co (**b**), and CNTs/Co (**c**)

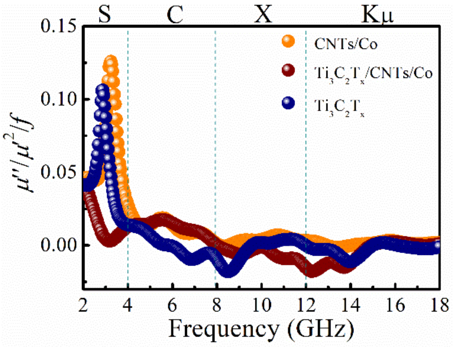

Fig. S15 Frequency-dependent $\mu''(\mu')^{-2}f^{-1}$ curves of $Ti_3C_2T_x$, $Ti_3C_2T_x$/CNTs/Co, and CNTs/Co

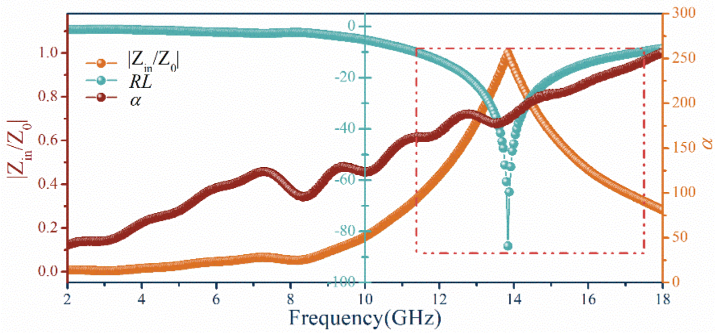

Fig. S16 Frequency-dependent $|Z_{in}/Z_0|$, α, and RL values of $Ti_3C_2T_x$/CNTs/Co-1.4 mm nanocomposites

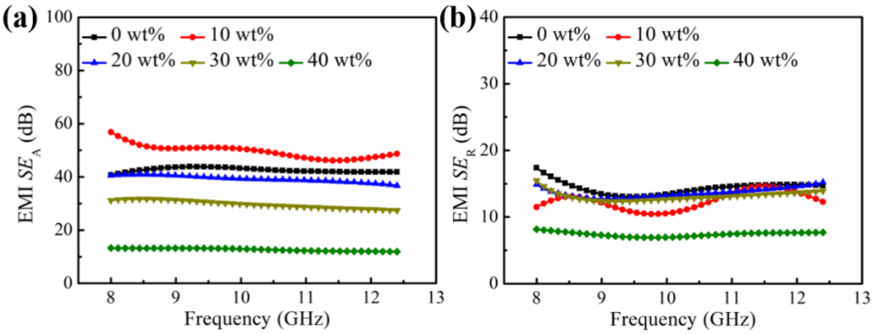

Fig. S17 EMI shielding measurements (SE_A (**a**) and SE_R (**b**)) of 40-μm-thick $Ti_3C_2T_x$/CNTs/Co nanocomposites with different content of CNTs/Co (0, 10, 20, 30, and 40 wt%)

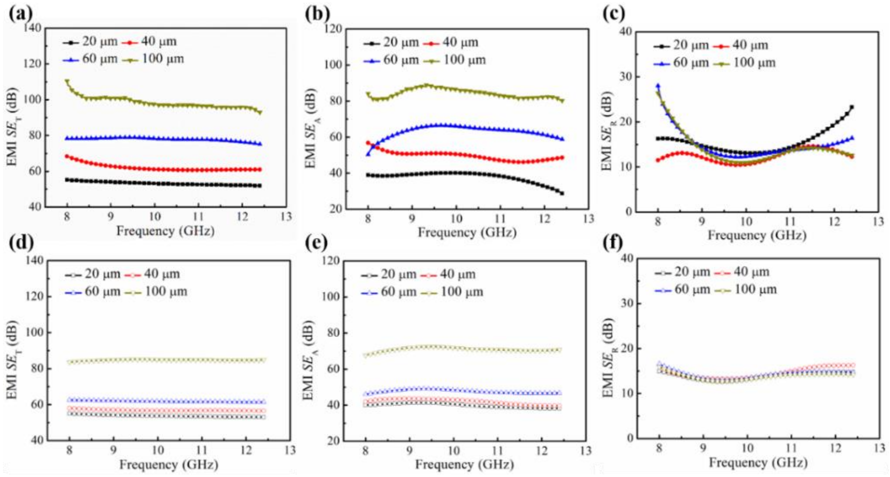

Fig. S18 EMI shielding measurements of $Ti_3C_2T_x$/CNTs/Co $_{(10\ wt\%)}$ (SE_T (**a**), SE_A (**b**) and SE_R (**c**)) and $Ti_3C_2T_x$ (SE_T (**d**), SE_A (**e**) and SE_R (**f**)) nanocomposites with different thickness (20, 40, 60, and 100 μm)

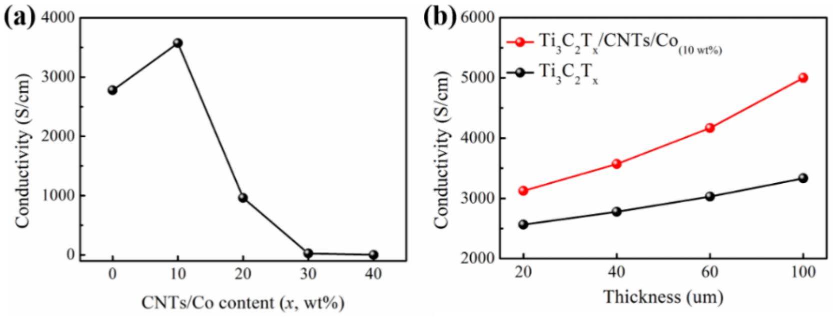

Fig. S19 (**a**) Conductivity of 40-μm-thick $Ti_3C_2T_x$/CNTs/Co with different content of CNTs/Co (0, 10, 20, 30, and 40 wt%). (**b**) The conductivity of $Ti_3C_2T_x$/CNTs/Co$_{(10\ wt\%)}$ nanocomposites with different thickness (20, 40, 60, and 100 μm)

Table S1 Electromagnetic wave absorption performance of the reported MXene-based composites

Sample	Filler loading (wt%)	EAB (GHz)	RL_{min} (dB)	*d* (mm)	Refs.
Ni/$Ti_3C_2T_x$/RGO aerogel	0.64	5.4	-75.2	2.15	[S1]
$Ti_3C_2T_x$/Ni chain/ZnO array cotton	/	4.2	-35.1	2.8	[S2]
$Ti_3C_2T_x$/gelatin aerogel	/	6.2	-59.5	2.0	[S3]
CF@$Ti_3C_2T_x$@MoS_2	20	7.6	-61.5	3.5	[S4]
Ni/$Ti_3C_2T_x$	10	3.7	-52.6	3.0	[S5]
$Ti_3C_2T_x$/GO aerogel	10	2.9	-49.1	1.2	[S6]
$Ti_3C_2T_x$/$NiCo_2O_4$	50	/	-51.0	2.2	[S7]
CoFe/$Ti_3C_2T_x$	60	2.6	-36.3	2.2	[S8]
MoS_2/TiO_2/$Ti_3C_2T_x$	50	2.6	-16.0	2.5	[S9]
$Ti_3C_2T_x$/Co	50	/	-46.5	1.0	[S10]
RGO/$Ti_3C_2T_x$	/	4.2	-20.0	3.2	[S11]
$Ti_3C_2T_x$/CNTs/Co	5	6.1	-85.8	1.4	This work

Table S2 EMI shielding efficiency of the reported MXene-based composites

Sample	Filler (wt %)	Matrix	*d* (μm)	*SE*(GHz)	Refs.
$Ti_3C_2T_x$@CNT hybrid	Bulk	/	100	60.5	[S12]
$Ti_3C_2T_x$	Bulk	/	45	92.0	[S13]
$Mo_2Ti_2C_3T_x$	Bulk	/	2.5	26.0	[S13]
Ti_3CNT_x	Bulk	/	40	116.2	[S14]
V_2CT_x			12	46.0	
Nb_2CT_x	Bulk	/	10	15.0	[S15]
Ti_2CT_x			11	50.0	
$Ti_3C_2T_x$/SA aerogel	6.1	PDMS	2000	53.9	[S16]
Ti_3C_2/SWCNT	/	PVA/PSS	0.2	3.4	[S17]
$Ti_3C_2T_x$ aerogel	Bulk	/	1000	44.8	[S18]
Fe_3O_4@$Ti_3C_2T_x$/ elastomer	15	DENR latex	1197	58.0	[S19]
$Ti_3C_2T_x$-AgNW	/	Epoxy resin	9000	49.2	[S20]
TiO_2-$Ti_3C_2T_x$/graphene	/	/	9.17	27.0	[S21]
$Ti_3C_2T_x$/CNF aerogel	Bulk	/	2000	74.6	[S22]
$Ti_3C_2T_x$/CNF film	Bulk	/	35	40.0	[S23]
			20	53.2	
$Ti_3C_2T_x$/CNTs/Co	Bulk	/	40	62.0	This work
			60	78.3	
			100	110.1	

Supplementary References

[S1] L. Liang, Q. Li, X. Yan, Y. Feng, Y. Wang et al., Multifunctional magnetic $Ti_3C_2T_x$ MXene/graphene aerogel with superior electromagnetic wave absorption performance. ACS Nano **15**(4), 6622-6632 (2021). https://doi.org/10.1021/acsnano.0c09982

[S2] S. Wang, D. Li, Y. Zhou, L. Jiang. Hierarchical $Ti_3C_2T_x$ MXene/Ni chain/ZnO array hybrid nanostructures on cotton fabric for durable self-cleaning and enhanced microwave absorption. ACS Nano **14**(7), 8634-8645 (2020). https://doi.org/10.1021/acsnano.0c03013

[S3] M. Yang, Y. Yuan, Y. Li, X. Sun, S. Wang et al., Anisotropic electromagnetic absorption of aligned $Ti_3C_2T_x$ MXene/gelatin nanocomposite aerogels. ACS Appl. Mater. Interfaces **12**(29), 33128-33138 (2020). https://doi.org/10.1021/acsami.0c09726

[S4] J. Wang, L. Liu, S. Jiao, K. Ma, J. Lv et al., Hierarchical carbon fiber@MXene@MoS_2 core-sheath synergistic microstructure for tunable and efficient microwave absorption. Adv. Funct. Mater. **30**, 2002595 (2020). https://doi.org/10.1002/adfm.202002595

[S5] L. Liang, R. Yang, G. Han, Y. Feng, B. Zhao et al., Enhanced electromagnetic wave-absorbing performance of magnetic nanoparticles-anchored 2D $Ti_3C_2T_x$ MXene. ACS Appl. Mater. Interfaces **12**(2), 2644-2654 (2020). https://doi.org/10.1021/acsami.9b18504

[S6] Y. Li, F. Meng, Y. Mei, H. Wang, Y. Guo et al., Electrospun generation of $Ti_3C_2T_x$

MXene@graphene oxide hybrid aerogel microspheres for tunable high-performance microwave absorption. Chem. Eng. J. **391**, 123512 (2020). https://doi.org/10.1016/j.cej.2019.123512

[S7] T. Hou, B. Wang, M. Ma, A. Feng, Z. Huang et al., Preparation of two-dimensional titanium carbide ($Ti_3C_2T_x$) and $NiCo_2O_4$ composites to achieve excellent microwave absorption properties. Compos. Part. B-Eng. **180**, 107577 (2020). https://doi.org/10.1016/j.compositesb.2019.107577

[S8] C. Zhou, X. Wang, H. Luo, L. Deng, S. Wang et al., Interfacial design of sandwich-like CoFe@$Ti_3C_2T_x$ composites as high efficient microwave absorption materials. Appl. Surf. Sci. **494**, 540-550 (2019). https://doi.org/10.1016/j.apsusc.2019.07.208

[S9] H. Wang, H. Ma. The electromagnetic and microwave absorbing properties of MoS_2 modified $Ti_3C_2T_x$ nanocomposites. J. Mater. Sci. **30**(16), 15250-15256 (2019). https://doi.org/10.1007/s10854-019-01897-7

[S10] F. Pan, L. Yu, Z. Xiang, Z. Liu, B. Deng et al., Improved synergistic effect for achieving ultrathin microwave absorber of 1D co nanochains/2D carbide MXene nanocomposite. Carbon **172**, 506-515 (2021). https://doi.org/10.1016/j.carbon.2020.10.039

[S11] X. Li, X. Yin, C. Song, M. Han, H. Xu et al., Self-assembly core-shell graphene-bridged hollow MXenes spheres 3D foam with ultrahigh specific EM absorption performance. Adv. Funct. Mater. **28**(41), 1803938 (2018). https://doi.org/10.1002/adfm.201803938

[S12] R. Yang, X. Gui, L. Yao, Q. Hu, L. Yang et al., Ultrathin, lightweight, and flexible cnt buckypaper enhanced using MXenes for electromagnetic interference shielding. Nano-Micro Lett. **13**(1), 66 (2021). https://doi.org/10.1007/s40820-021-00597-4

[S13] F. Shahzad, M. Alhabeb, C. B. Hatter, B. Anasori, S. M. Hong et al., Electromagnetic interference shielding with 2D transition metal carbides (MXenes). Science **353**(6304), 1137-1140 (2016). https://doi.org/10.1126/science.aag2421

[S14] A. Iqbal, F. Shahzad, K. Hantanasirisakul, M.-K. Kim, J. Kwon et al., Anomalous absorption of electromagnetic waves by 2D transition metal carbonitride Ti_3CNT_x (MXene). Science **369**(6502), 446-450 (2020). https://doi.org/10.1126/science.aba7977

[S15] M. Han, C.E. Shuck, R. Rakhmanov, D. Parchment, B. Anasori et al., Beyond $Ti_3C_2T_x$: MXenes for electromagnetic interference shielding. ACS Nano **14**(4), 5008-5016 (2020). https://doi.org/10.1021/acsnano.0c01312

[S16] X. Wu, B. Han, H.-B. Zhang, X. Xie, T. Tu, et al., Compressible, durable and conductive polydimethylsiloxane-coated MXene foams for high-performance electromagnetic interference shielding. Chem. Eng. J. **381**, 122622 (2020). https://doi.org/10.1016/j.cej.2019.122622

[S17] G. M. Weng, J. Li, M. Alhabeb, C. Karpovich, H. Wang et al., Layer-by-layer assembly of cross-functional semi-transparent MXene-carbon nanotubes composite films for next-generation electromagnetic interference shielding. Adv. Funct. Mater. **28**(44), 1803360 (2018). https://doi.org/10.1002/adfm.201803360

[S18] M. Han, X. Yin, K. Hantanasirisakul, X. Li, A. Iqbal et al., Anisotropic MXene aerogels with a mechanically tunable ratio of electromagnetic wave reflection to absorption. Adv. Opt. Mater. **7**(10), 1900267 (2019). https://doi.org/10.1002/adom.201900267

[S19] Q. Song, B. Chen, Z. Zhou, C. Lu. Flexible, stretchable and magnetic Fe_3O_4@$Ti_3C_2T_x$/elastomer with supramolecular interfacial crosslinking for enhancing mechanical and electromagnetic interference shielding performance. Sci. China Mater. **64**, 1437-1448 (2021). https://doi.org/10.1007/s40843-020-1539-2

[S20] W. Chen, L.-X. Liu, H.-B. Zhang, Z.-Z. Yu. Flexible, transparent, and conductive $Ti_3C_2T_x$ MXene-silver nanowire films with smart acoustic sensitivity for high-performance electromagnetic interference shielding. ACS Nano **14**(12), 16643-16653 (2020). https://doi.org/10.1021/acsnano.0c01635

[S21] C. Xiang, R. Guo, S. Lin, S. Jiang, J. Lan et al., Lightweight and ultrathin TiO_2-$Ti_3C_2T_x$/graphene film with electromagnetic interference shielding. Chem. Eng. J. **360**, 1158-1166 (2019). https://doi.org/10.1016/j.cej.2018.10.174

[S22] Z. Zeng, C. Wang, G. Siqueira, D. Han, A. Huch et al., Nanocellulose-MXene biomimetic aerogels with orientation-tunable electromagnetic interference shielding performance. Adv. Sci. **7**(15), 2000979 (2020). https://doi.org/10.1002/advs.202000979

[S23] B. Zhou, Z. Zhang, Y. Li, G. Han, Y. Feng et al., Flexible, robust, and multifunctional electromagnetic interference shielding film with alternating cellulose nanofiber and MXene layers. ACS Appl. Mater. Interfaces **12**(4), 4895-4905 (2020). https://doi.org/10.1021/acsami.9b19768
